# A mathematical modelling tool for unravelling the antibody-mediated effects on CTLA-4 interactions

**DOI:** 10.1186/s12911-018-0606-x

**Published:** 2018-06-11

**Authors:** Aravindhan Ganesan, Theinmozhi Arulraj, Tahir Choulli, Khaled H. Barakat

**Affiliations:** 1 Faculty of Pharmacy and Pharmaceutical Sciences, University of Alberta, Edmonton, Canada; 2 School of Life Sciences, University of Hyderabad, Hyderabad, India; 3 Department of Mathematical and Statistical Sciences, Faculty of Science, University of Alberta, Edmonton, Canada; 4 Li Ka Shing Institute of Virology, University of Alberta, Edmonton, Canada

**Keywords:** CTLA-4, Immune checkpoints, Ipilimumab, Tremelimumab, Antibody, Mathematical modeling

## Abstract

**Background:**

Monoclonal antibodies blocking the Cytotoxic T-lymphocyte antigen 4 (CTLA-4) receptor have revolutionized the field of anti-cancer therapy for the last few years. The human T-cell-based immune responses are modulated by two contradicting signals. CTLA-4 provides a T cell inhibitory signal through its interaction with B7 ligands (B7–1 and B7–2), while CD28 provides a stimulatory signal when interacting with the same ligands. A previous theoretical model has focused on understanding the processes of costimulatory and inhibitory complex formations at the synapse. Nevertheless, the effects of monoclonal antibody (mAb)-mediation on these complexes are relatively unexplored. In this work, we expand on the previous model to develop a new mathematical framework for studying the effects of anti-CTLA-4 mAbs on the co-stimulatory (CD28/B7 ligands) and the co-inhibitory (CTLA-4/B7 ligands) complex formation at the immunological synapse. In particular, we focus on two promising anti-CTLA-4 mAbs, tremelimumab (from AstraZeneca) and ipilimumab (from Bristol-Myers Squibb), which are currently in clinical trials and the market, respectively, for targeting multiple tumors.

**Methods:**

The mathematical model in this work has been constructed based on ordinary differential equations and available experimental binding kinetics data for the anti-CTLA-4 antibodies from literature.

**Results:**

The numerical simulations from the current model are in agreement with a number of experimental data. Especially, the dose-curves for blocking the B7 ligand binding to CTLA-4 by ipilimumab are comparable with the results from a previous competitive binding assay by flow cytometry and ELISA. Our simulations predict the dose response and the relative efficacies of the two mAbs in blocking the inhibitory CTLA-4/B7 complexes.

**Conclusions:**

The results show that different factors, such as multivalent interactions, mobility of molecules and competition effects, could impact the effects of antibody-mediation. The results, in particular, describe that the competitive effects could impact the dose-dependent inhibition by the mAbs very significantly. We present this model as a useful tool that can easily be translated to study the effects of any anti-CTLA-4 antibodies on immunological synaptic complex formation, provided reliable biophysical data for mAbs are available.

**Electronic supplementary material:**

The online version of this article (10.1186/s12911-018-0606-x) contains supplementary material, which is available to authorized users.

## Background

**Fig. 1:**
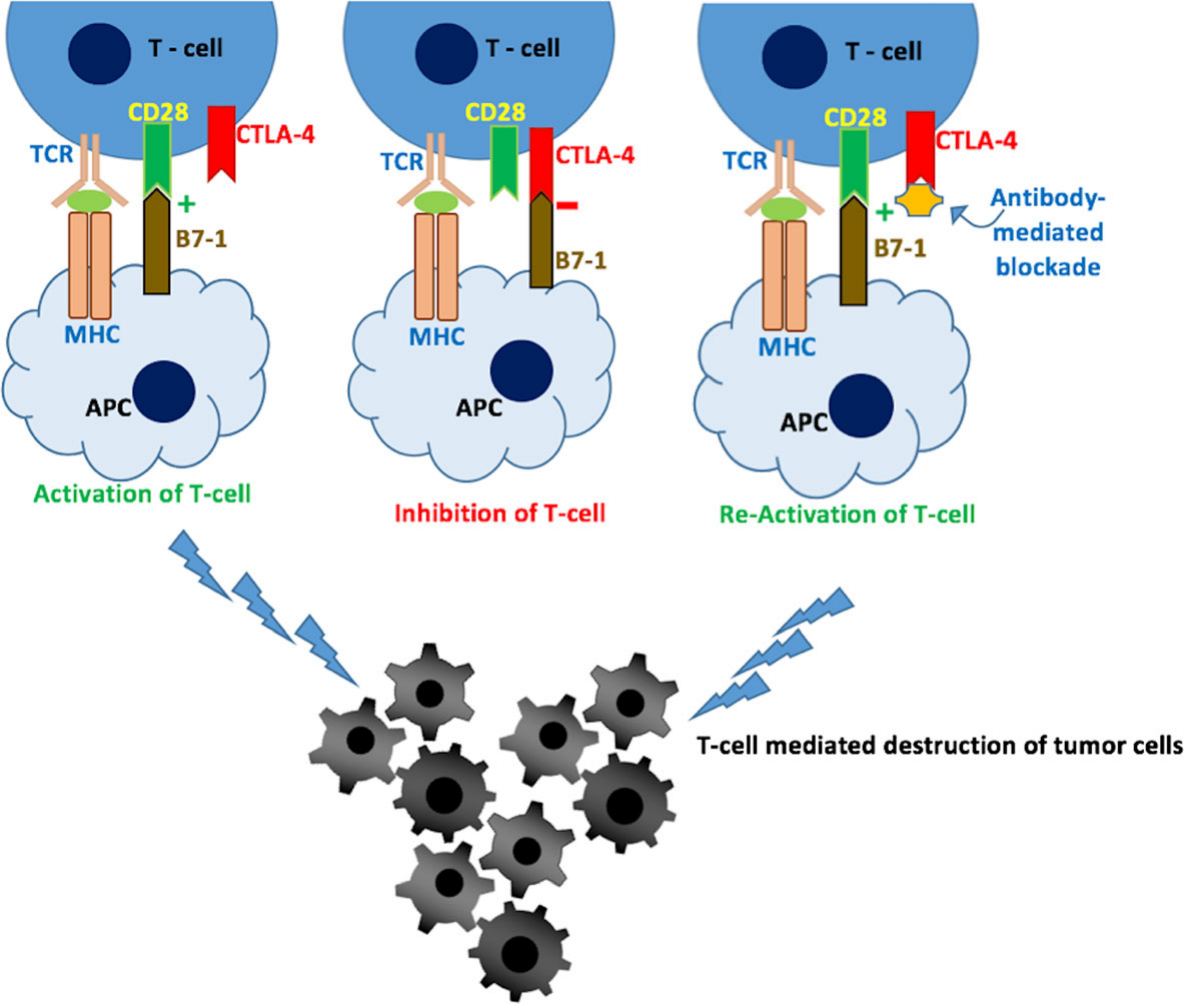
Schematic representation of T-cell activation and inhibition by the interactions of CD28 and CTLA-4 with the B7 ligands, B7–1 and B7–2, respectively

Blockade of immune checkpoints has recently been proven as a revolutionary strategy in the fight against cancers [1–6]. T-cells play a pivotal role in modulating the immune response against pathogens [7] and also in the prevention of autoimmunity [6]. According to the classic ‘two-signal’ model in immunology [8, 9], T-cell-mediated immune responses are activated by two different protein-protein interactions taking place at the immunological synapse [1–5, 10–12] (Fig. 1). The first signal is triggered when the T-cell receptors (TCRs) recognize the major histocompatibility complex (MHC) on the surface of antigen-presenting cells (APCs). A second signal comes from the binding of CD28, a co-receptor expressed on the T-cell surface, with its’ complementary B7 ligands, known as B7–1 (or CD80) and B7–2 (or CD86), on the surface of APCs. This second signal is required to activate and sustain the activity of T-cells [3, 5, 13, 14]; hence it is known as a co-stimulatory signal and CD28 is dubbed as a co-stimulatory receptor (Fig. 1). On the other hand, there are a number of negative signal stimuli (known as the inhibitory receptors) that act to inactivate the T-cells, including cytotoxic T-lymphocyte-associated protein-4 (CTLA-4 or CD152) and programmed death 1 (PD1 or CD279) [3]. Particularly, CTLA-4, a CD28 homologue (with ~ 30% sequence identity) expressed on the T-cell surface, binds with the same set of B7-ligands of CD28; albeit with higher affinity towards B7–1 and B7–2. Both CD28 and CTLA-4 are transmembrane proteins of the immunoglobulin superfamily that exist as homodimers [15] .Nevertheless, CD28 is known to bind the ligands monovalently; whereas, CTLA-4 is bivalent in nature that, infact, allows it to bind the B7 ligands with high avidity [15, 16]. Given such differences, under competitive environments, CTLA-4 would be able to out-compete CD28 for ligand-binding [17]. The functionalities of CD28 and CTLA-4 are interlinked and both co-stimulatory and co-inhibitory signals are required for maintaining the immunological balance between self-tolerance and defending against foreign entities and pathogens under normal conditions [4–6, 18, 19].

Cancer cells are able to escape the immunological surveillance of T-cells, by overexpressing the inhibitory receptors that attenuate anti-tumor immune response [4, 5]. As a result, in principal, blocking the co-inhibitory receptor-ligand interactions (schematically shown in Fig. 1) should be able to facilitate the re-activation of T-cells, which in-turn will recognize and eliminate cancers. Monoclonal antibodies (mAbs) blocking inhibitory immune checkpoints have demonstrated exceptional therapeutic benefits in clinical trials [20–22], which is transforming human cancer treatment. Ipilimumab, a completely human IgG1 antibody from Bristol-Myers Squibb, became the first-in-class anti-CTLA-4 mAb to be approved by the US Food and Drug Administration (FDA) in 2011 for the treatment of metastatic melanoma [23, 24]. Several clinical trials showed that monotherapy with ipilimumab in metastasis melanoma patients increased the overall responsive rate by 10–20% [1, 25]. When combined with other immune checkpoint therapies, ipilimumab is able to offer much enhanced benefits [26, 27]. Another IgG2 anti-CTLA-4 mAb, Tremelimumab from AstraZeneca, is currently in phase III clinical trials [1, 5]. Similar to Ipilimumab, AstraZeneca’s tremelimumab binds specifically to CTLA-4 and blocks its interaction with the B7 ligands. Tremelimumab is also being tested in combination with other immune checkpoint mAbs for targeting multiple tumors [28, 29], including melanoma, colon cancer, and mesothelioma [28, 29]. The progress made by these two antibodies, from bench to final clinical trial phases or to the market, has boosted the interests towards developing more promising immune-checkpoint blocking inhibitors (both mAbs and, lately, small molecules).

Very recently, He et al. [1] investigated the binding profiles of both mAbs against CTLA-4 using surface plasmon resonance (SPR) experiments and reported their K_D_ values as 18.2 nM for ipilimumab/CTLA-4 complex and 5.89 nM for tremelimumab/CTLA-4 complex. These binding affinity values are higher than (or, in some cases, comparable to) those reported for B7–1 ligands with CTLA-4 [1]. However, it is important to note that different range of affinity values have been reported for the CTLA-4/B7–1 complexes. For example, an earlier study reported that a soluble B7–1 Ig fusion protein bound to CTLA-4 with a dissociation constant of ~ 12 nM [30]. However, this value was argued to be higher when compared with other protein-protein interactions occurring between T-cell surface and APCs [31]. The authors of this work [30] had described that the CTLA-4Ig in their experiments was not monomeric in solution, and possibly formed higher aggregates that might have possibly resulted in the high apparent K_D_ values for the interactions of CTLA-4Ig/B7Ig fusion protein. Another study [31] based on SPR experiments reported that, at 37 °C, soluble recombinant B7–1 bound to CTLA-4 with a K_D_ value of 0.42 μM. This indicates that affinity between B7–1 and CTLA-4 is relatively lower than that of ipilimumab/CTLA-4 and tremelimumab/CTLA-4 complexes. The association rate constant (k_a_) of ipilimumab (3.83 × 10^5^/Ms) and tremelimumab (3.08 × 10^5^/Ms) are almost close to each other, however, their dissociation rate constants (k_d_) were significantly different [1]. The k_d_ value for ipilimumab was 6.96 × 10^− 3^/s, whereas for tremelimumab it was 1.8 × 10^− 3^/s [1]. This clearly indicates that the tremelimumab is able to form much stable complex with CTLA-4 when compared to that of ipilimumab. Thus, understanding the effects of these two potent anti-CTLA-4 antibodies on the co-stimulatory and the co-inhibitory complex formation at the synapse will be useful to develop effective next-generation anti-CTLA-4 therapeutics. The ultimate objective of this work is to precisely study these effects using mathematical modelling and simulations.

Mathematical modelling and simulation remains a powerful tool to gain quantitative insights into the dynamics of complicated systems [32–40]. Especially, mathematical modelling is gaining more popularity in the field of cancer immunotherapy. For example, Kirschner et al. [38] developed a mathematical model, based on ordinary differential equations (ODEs) to simulate the dynamics between tumor cells, immune-effector cells and cytokine interleukin-2. This model was useful to explore the effects of adoptive cellular immunotherapy (ACI) on the model and found that a combination therapy with ACI and IL-2 could boost the immune system sufficiently to clear the tumor [38]. Krnoik et al. [37] developed an expanded mathematical model, from a previously published model, in order to simulate cellular immunotherapy in melanoma. This model was useful to understand the findings from clinical trials suggesting that patients with the smallest tumor load respond better for this kind treatment [37]. Mathematical models have also been useful to understand tumor growth, response to therapy and the interactions of immune cells with the cancer cell [39–42]. Bidot et al. [43] developed a mathematical model for studying the kinetics of monoclonal T-cell-specific activation. This model attempted to account for the sequence of events starting from the TCR-MHC binding to T-cell activation and response [43]. Mathematical modelling has also been employed to study the complex formation between PD-1 receptor and its ligands [44]. Jansson et al. [33] developed a theoretical model for simulating the synaptic accumulation of the molecules involved in co-stimulation and inhibition of T-cells. This model, which was developed based on the Ordinary Differential Equations (ODEs) and rigorous biophysical and expression data from literature, explained the interactions of CTLA-4 and CD-28 with their B7-ligands in the context of a potentially dynamic synaptic microenvironment [33]. However, until now, there is no model that could predict the response to CTLA-4 blocking antibodies and the consequent effects on complex formation at immunological synapse.

In this work, we expand the model of Jansson et al. [33] by including the effect of an anti-CTLA-4 antibody. Our model is based on ODEs and additional biophysical data for antibody-CTLA-4 complexes [1]. Using this new model, we studied how the binding of mAbs (with different affinities) to CTLA-4 dynamically changed the interactions among co-inhibitory complexes (CTLA-4/B7–1 and CTLA-4/B7–2) and co-stimulatory complexes (CD28/B7–1 and CD28/B7–2) at the immunological synapse. The numerical simulations from the model have been validated by different experimental data reported earlier. This model should be a useful tool to predict the dose response of any anti-CTLA-4 antibodies and their impacts on synaptic complex formation processes.

## Methods

### Mathematical modelling assumptions

As mentioned above, the mathematical model in this work is based on the previous two-compartment model by Jansson et al. [33], which was constructed to simulate the CD28 and CTLA-4 complex formation with the B7-ligands at immunological synapses. In this study, we developed an expanded model to assess the changes in the co-stimulatory (CD28/B7 ligands) and the co-inhibitory (CTLA-4/B7 ligands) interactions upon binding of the antibody to the free CTLA-4 receptor sites. This model, as in Jansson’s model [33], involves two components that includes the synapse and the region outside of the synapse. It is assumed that CD28, B7–1 and B7–2 are primarily unbound and distributed uniformly over the surface. CTLA-4, on the other hand, is present intracellularly and gets injected into the synapse upon activation [45, 46]. The ‘free-diffusion’ model has been applied to control the mobility of the molecules, such that only the mobile molecules are able to diffuse into the synapse, while the immobile species outside of the synapse are ignored. It is understandable that the immobile species outside of the synapse are, anyway, not able to participate in any complex formation. On the other hand, the immobile molecules inside the synapse stay there and are involved in the protein-protein interactions. The model also assumes that the CTLA-4 receptors, once injected from the intracellular environment, stay within the synapse. In the case of antibody, unlike the membrane-bound ligands, it is in the free solution and is assumed to bind to free CTLA-4 monovalently. The model assumes that the binding site of B7–1, B7–2 and the antibody molecules on CTLA-4 overlap considerably [2], and, therefore, only one of them (B7–1 or B7–2 or the antibody) is able to bind with a CTLA-4 molecule. Moreover, in our model, the antibody is allowed to bind an unbound CTLA-4 monomer that is part of a dimer, where the other monomer can be bound with a B7 ligand. Due to the lack of parameters for the association and dissociation of complexes in the model, rate constants from similar complexes are used and reactions are modelled as parallel mass action reactions. Bivalent association and dissociation rate constants are employed for the binding/unbinding of B7 ligands to a CTLA-4 monomer that is part of a dimer, where the other monomer is bound to an antibody.

### Parameters

The parameters for the interactions of CTLA-4 and CD28 with the B7 ligands in the current model are almost similar to those in Jansson’s model [33], which are given in the supplementary information (Additional file 1: Table S1). However, the association and dissociation rate constants for the interactions of antibodies, tremelimumab and ipilimumab, with CTLA-4 receptor are collected from the literature [1] and employed in the current model. The reported SPR experiments [1] measured the association rate constants of ipilimumab and tremelimumab with CTLA-4 to be 3.83 × 10^5^/Ms. and 3.08 × 10^5^/Ms., respectively. Whereas, their dissociation rate constants were observed to be 6.96 × 10^− 3^/s (ipilimumab) and 1.8 × 10^− 3^/s [1] (tremelimumab). It is important to note that the rate constants for the interactions of antibodies are not converted to 2-D rates, as the antibodies are present in the solution. This approximation is reasonable, when the binding site is accessible [47].

### Antibody complex formation

The equations related to the modelling of rates of change for the complex formation between different species, such as CD28, CTLA-4, B7–1 and B7–2, are all similar to those of Jansson et al. [33]. However, a number of additional terms are employed in the current model in order to account for the increase or decrease in density of these species in response to the association and dissociation of antibodies to that of CTLA-4 monomers. Please note that the rate of change of the density of complexes for the newly added terms in the model are also written using the mass-action law.

### Rate of change of density of antibody/CTLA-4 complex

The antibody associates with a CTLA-4 dimer at the rate of k_on_, in order to form the antibody/(CTLA-4)_2_ complex (referred as Ac complex in the equation). The rate for dissociation of antibody from the Ac complex is k_off_. An unbound B7–1 associates with the Ac complex at the rate of α_44_to form a complex of Ac/B7–1 (or EAb_1_), whereas, an unbound B7–2 associates with the Ac complex at the rate of α_33_ to form Ac/B7–2 (or Ab/CTLA4/B72). An antibody binds to the Ac complex at the rate of Kon to form the resultant Ab/Ac (or AcA) complex. The association of either of the B7–1 ligands or antibody to the Ac complex reduces the density of the latter. On the other hand, dissociation of B7–1 (rate = δ_44_), B7–2 (rate = δ_33_) and antibody (rate = k_off_) from the EAb1, Ab/CTLA4/B72, AcA complexes, respectively, increases the density. Hence, the rate of change in the density of the Ac complex can be written as follows,

1 $$ {\displaystyle \begin{array}{l}\frac{dAc}{dt}=-{\alpha_{44}}^{\ast }{Ac}^{\ast }B71+{\delta_{44}}^{\ast }{EAb}_1-{k_{on}}^{\ast }{Ac}^{\ast } Ab+{k_{off}}^{\ast } Ac A+{k_{on}}^{\ast } CTLA{4}^{\ast } Ab\\ {}-{k_{off}}^{\ast } Ac-{\alpha_{33}}^{\ast }{Ac}^{\ast }B72+{\delta_{33}}^{\ast } Ab/ CTLA4/B72\end{array}} $$

The rate of change of density for the other antibody-mediated complexes are given in supplementary information, Additional file 2: Table S2. The numbers of free antibody molecules is increased by the dissociation of mAb from any of the antibody-included complexes (such as, Ac, AcA, Ab/CTLA-4/B72, EAb, CAb, DAb). On the other hand, association of free antibody to form any of the above complexes decreases the total number of free antibody molecules,and this rate of change in the antibody can be written as,

2 $$ {\displaystyle \begin{array}{l}\frac{dAb}{dt}=-{k_{on}}^{\ast } CTLA{4}^{\ast } Ab+{k_{off}}^{\ast } Ac-{k_{on}}^{\ast }{Ac}^{\ast } Ab+{k_{off}}^{\ast } Ac A\\ {}-{k_{on}}^{\ast }{Ab}^{\ast}\sum \limits_{k=1}^{\infty }{E}_k+{k_{off}}^{\ast}\sum \limits_{k=1}^{\infty }{EAb}_k-{k_{on}}^{\ast }{Ab}^{\ast}\sum \limits_{k=1}^{\infty }{C}_k+{k_{off}}^{\ast}\sum \limits_{k=1}^{\infty }{CAb}_k-{k_{on}}^{\ast }{Ab}^{\ast}\sum \limits_{k=1}^{\infty }{CAb}_k+{k_{off}}^{\ast}\sum \limits_{k=1}^{\infty }{DAb}_k\\ {}-{k_{on}}^{\ast } CTLA4/B{72}^{\ast } Ab+{k_{off}}^{\ast } Ab/ CTLA4/B72\end{array}} $$

Density of mobile CD28, B71 and B72 molecules, present outside synapse is estimated by subtracting the total number of mobile molecules of CD28, B71,B72 that are part of complexes formed at synapse.

3 $$ CD28 out={t_{CD28, tot}}^{\ast }{m}_{CD28}-\left( CD28+ CD28/B71+{2}^{\ast }{(CD28)}_2/B71+ CD28/B72-{i}_{CD28}\right)\ast {a}_{syn} $$

4 $$ {\displaystyle \begin{array}{l}B71 out={t_{B71, tot}}^{\ast }{m}_{B71}-\left(B71+ CD28/B71+{(CD28)}_2/B71\right.\\ {}\left.+\sum \limits_{k=1}^{\infty }{B}_k\left(k+1\right)+\sum \limits_{k=1}^{\infty }{C}_kk+\sum \limits_{k=1}^{\infty }{E}_kk+\sum \limits_{k=1}^{\infty }{EAb}_kk+\sum \limits_{k=1}^{\infty }{CAb}_kk+\sum \limits_{k=1}^{\infty }{DAb}_kk-{i}_{B71}\right)\ast {a}_{syn}\end{array}} $$

5 $$ {\displaystyle \begin{array}{l}B72 out={t_{B72, tot}}^{\ast }{m}_{B72}-\left(B72+ CD28/B72+ CTLA4/B72\right.\\ {}\left.+{2}^{\ast } CTLA4/{(B72)}_2+ Ab/ CTLA4/B72-{i}_{B72}\right)\ast {a}_{syn}\end{array}} $$

Similarly, the number of CTLA4 molecules present inside the cell is calculated by subtracting it from total number of CTLA4 molecules.

6 $$ {\displaystyle \begin{array}{l} CTLA4\operatorname{int}={t_{CTLA4, tot}}^{\ast }{m}_{CTLA4}-\Big( CTLA4+ CTLA4/B72+ CTLA4/{(B72)}_2\operatorname{}\\ {}\operatorname{}+\sum \limits_{k=1}^{\infty }{B}_kk+\sum \limits_{k=1}^{\infty }{C}_k\left(k+1\right)+\sum \limits_{k=1}^{\infty }{E}_kk+\sum \limits_{k=1}^{\infty }{EAb}_kk+\sum \limits_{k=1}^{\infty }{CAb}_k\left(k+1\right)+\sum \limits_{k=1}^{\infty }{DAb}_k\left(k+1\right)+ Ab/ CTLA4/B72+ Ac+ Ac A\Big)\ast {a}_{syn}\end{array}} $$

### Modelling and simulations

Mathematical modelling and simulation procedures in this study were programmed using MATLAB software from MathWorks (https://www.mathworks.com/products/matlab.html). A stiff ordinary differential equation solver, ode15s, was used for solving the equations in the current model. The different components included in the model and their abbreviations are provided in the supplementary information (Additional file 3: Table S3). Each simulation was performed for 7 h (i.e., 25,200 s) unless otherwise stated. Initially, the simulations are performed with ‘0’ concentration of antibody, in order to reproduce the results from Jansson’s model [33]. Later, the simulations were performed with different concentrations of tremelimumab and ipilimumab and the effects on CTLA-4 and CD28 complex formation were analyzed.

## Results

### Simulation of antibody-free complex formation

**Fig. 2:**
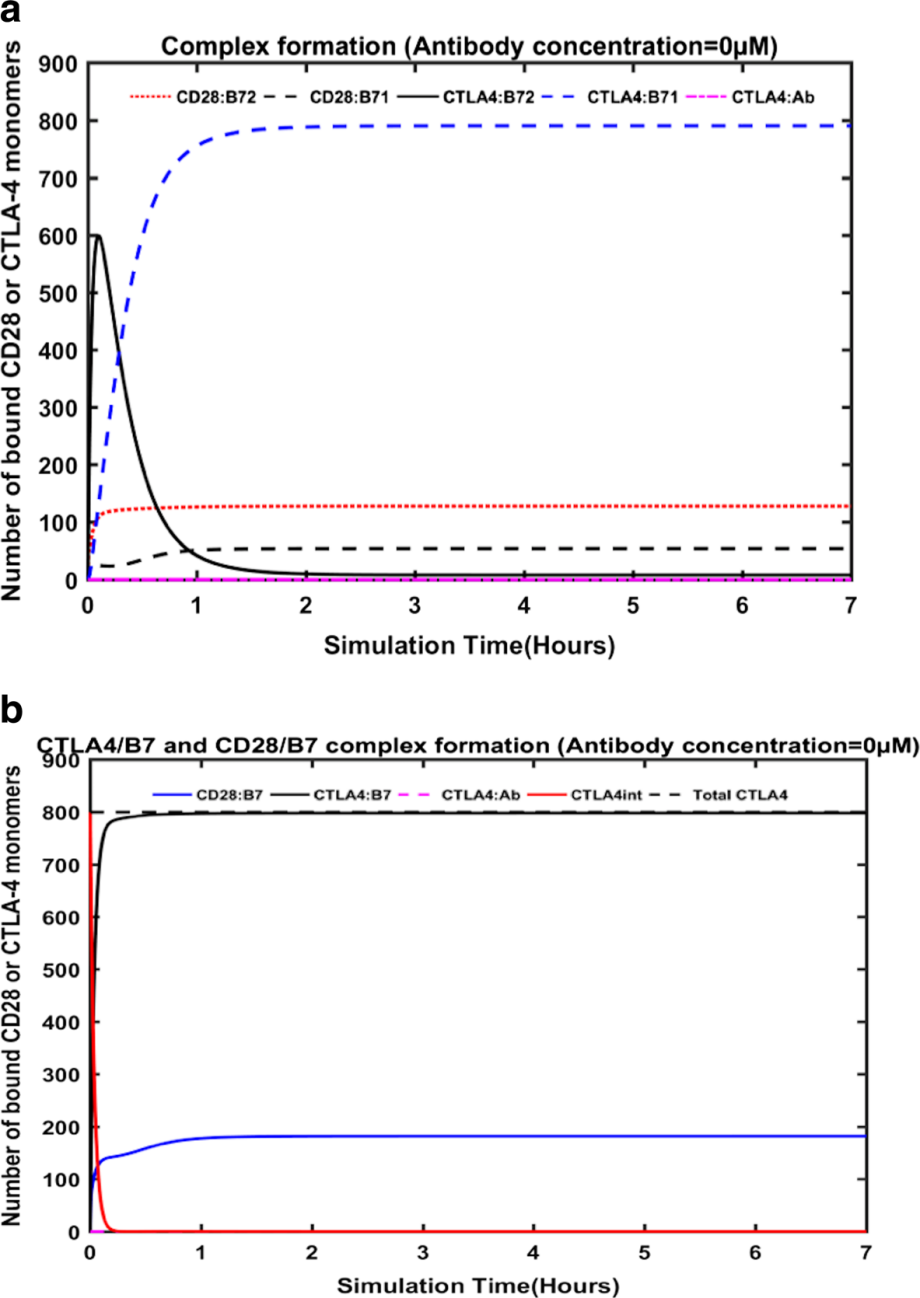
Complex formation in the free-diffusion model from the antibody-free simulations. The number of CD28 and CTLA-4 molecules bound to B7–1 and B7–2 at the immunological synapse, during the antibody-free simulations (**a**). The dynamic changes in the total numbers of CTLA-4/B7 complex, CD28/B7 complex, amount of CTLA-4 at the intra-cellular environment and the total number of CTLA-4 monomers are provided (**b**)

Initially, the simulation based on the free-diffusion model (Fig. 2a) was performed for ~ 7 h, without the inclusion of any antibody (i.e., 0 μM antibody concentration).This situation is ideally to reproduce the simulation from Jansson et al. [33].This simulation mimics an activated T-cell environment, where both CD28 and CTLA-4 are present at the synapse and are competing for ligand-binding. During the initial hours (~ 15 min), the expression levels of B7–2 at synapse is much higher and hence, it remained the leading ligand for CTLA-4 and CD28. Particularly, at about 6 min, the CTLA-4/B72 interactions reached a peak with ~ 600 CTLA-4 monomers were bound to B7–2, against ~ 140 CTLA-4/B7–1 and 108 CD28/B7–2 complexes. The domination of CTLA-4/B7–2 remained until ~ 17 min, however, this number (of CTLA-4/B7–2 complex) dropped significantly to < 50 at ~ 1 h of simulation. Gradually, as more CTLA-4 moved from the intracellular region to the synapse (see in Fig. 2b), the CTLA-4/B7–1 complex replaced CTLA-4/B7–2 and became the most populated complex after ~ 20 min. After reaching the steady state, it can be seen (in Fig. 2a) that the majority of the B7–1 was bound to CTLA-4 (resulting ~ 790 CTLA-4/B7–1 complexes), while only small numbers of B7–1 (~ 50) were engaged with the CD28 receptor. On the other hand, the amount of CD28/B7–2 complexes (~ 127) outnumbers the complexes formed by CTLA-4 monomers and B7–2(~ 8). As Jansson et al. [33] noted, these behaviors in the simulation are in good agreement with previous findings that B7–1 is the preferred ligand for CTLA-4 and B7–2 preferentially recruits CD28 at the synapse [48, 49]. Hence, 99% of CTLA-4 are complexed with B7–1, while only 1% of the receptor is engaged with the B7–2 ligand. Overall, however, the percentage of CTLA-4 complexed with the B7 ligands is much higher than that of CD28/B7 complexes (Fig. 2b). This trend is expected as CTLA-4 has a higher affinity towards B7 ligands than that of CD28.

### Binding of the antibodies to CTLA-4

**Fig. 3:**
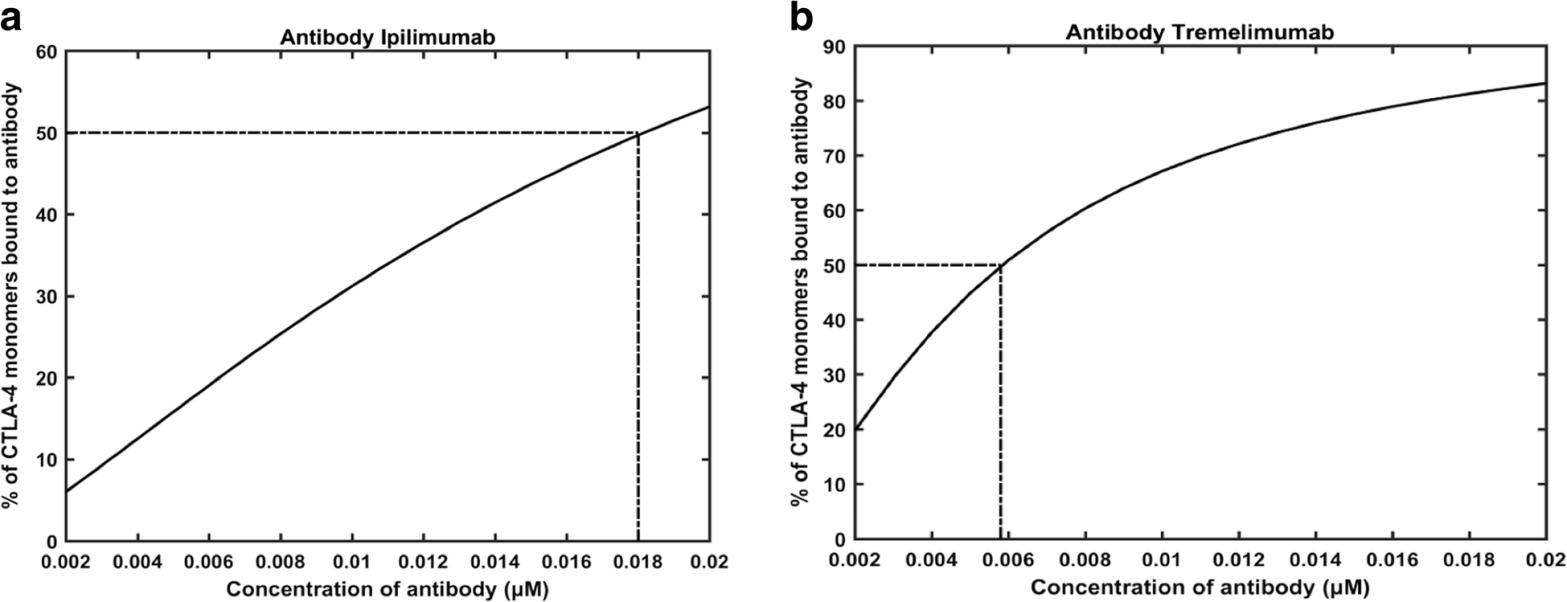
The reproduction of the experimental K_D_ values for the binding of CTLA-4 to ipilimumab (**a**) and tremelimumab (**b**)

Initially, we performed two simulations with an in silico knockout model, where CD28 and the B7 ligands (both B7–1 and B7–2) were muted and only CTLA-4 was allowed to bind with the antibodies, ipilimumab and tremelimumab, at different concentrations (0.002 μM to 0.018 μM). These simulations were carried out in order to confirm that, in a non-competitive setting, 50% of CTLA-4 had formed complex with the antibodies when the concentration of the antibodies equaled their respective K_D_ values. As shown in Figs. 3, 50% of the CTLA-4 monomers were bound to antibody at the concentrations of 0.018 μM and 0.0058 μM of ipilimumab (Fig. 3a) and tremelimumab (Fig. 3b), respectively. These concentrations are approximately close to the K_D_ values for these antibodies (ipilimumab – 18.2 nM; Tremelimumab – 5.89 nM), as reported by He et al. [1] based on their SPR experiments. Nevertheless, since the association and dissociation rate constants of the antibodies for the current model was obtained from the work of He et al. [1], it is expected that the model is able to achieve 50% complex formation at the concentration of the K_D_ values of mAbs. Hence, this confirms that the model simulates the complex formation correctly.

### Competitive binding of the antibodies to CTLA-4 and model validation

**Fig. 4:**
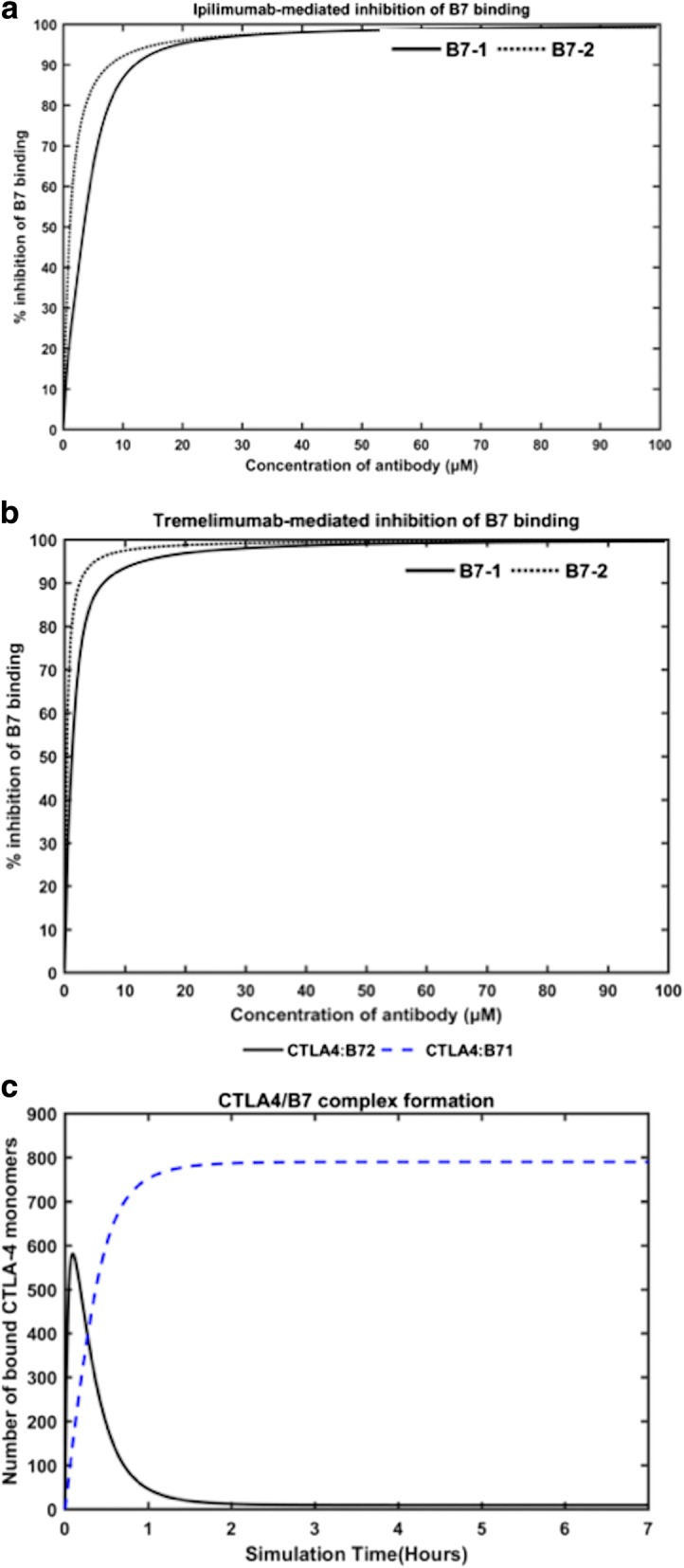
The dose-curves for the blocking of B7 ligands to CTLA-4 by ipilimumab (**a**) and tremelimumab (**b**) in the simulations performed in either of the B7 -knock-out model, where either of the B7 ligands and CD28 were removed. The complex formation of CTLA-4/B7–1 and CTLA-4/B7–2 in the absence of CD28 and antibody is also simulated (**c**)

Next, we performed the simulations to study how the antibodies, in the absence of CD-28, competed with either B7–1 or B7–2 for binding to CTLA-4. In order to perform these simulations, CD-28 and either of the B7 ligands were muted in the model. The resulting dose response curves for the percent inhibition of B7–1 binding and B7–2 binding with CTLA-4 at different concentrations of the antibodies, as predicted by the model, are shown in Fig. 4a and b, respectively. As shown in Fig. 4a and b, the model was able to simulate the dose-dependent inhibition of CTLA-4/B7 interactions by the competitive binding of ipilimumab and tremelimumab.

The dose-curves for blocking B7–1 and B7–2 binding by ipilimumab (Fig. 4a) is comparable with the experimental dose curves reported by Keler et al. [50] for the same antibody. It is important to note that no values from this experimental work have been used in our simulations or in the construction of our model. In this previous work [50], the scientists at Medarex performed competitive binding assay by flow cytometry and ELISA to demonstrate the ability of ipilimumab (dubbed as 10D1 in the paper) [50] to block the interactions of CTLA-4 with B7–1 and B7–2, separately. The simulations based on our model was also carried out by having only one of the B7 ligands active at a time, so as to mimic the experimental set-up [50]. Although the overall trends in the dose-response curves for ipilimumab obtained from our model and the previous experiment [50] are in agreement, the percent inhibition predicted for each dose of the antibody by our model are higher than those observed in the experiments [50]. For example, our model predicts that, at 10 μM concentration of ipilimumab, ~ 89% of B7–1 and ~ 92% of B7–2 are blocked; the experiments, on the other hand, reported ~ 70% and ~ 90% inhibition of B7–1 and B7–2 binding [50], respectively. Nevertheless, it should be noted that the previous experiments [50] were carried out with a human CTLA-4 (hCTLA-4) expressing cell, which was constructed by a hCTLA-4/murineCD3 chimeric gene. Thus, taking into account this difference and other experimental conditions, in general, the predictions from our model and the experiments are in reasonable agreement. Particularly, Keler et al. [50] reported an IC_50_ value of ~ 1–3 μM for ipilimumab (or 10D1 as named in the paper) to block the B7 ligands, which is in excellent agreement with the values predicted by our model (IC_50_ = 1.11 μM for B7–2 blocking; and 3.5 μM for B7–1 blocking). This validates the ability of our model to simulate the competitive binding between antibodies and the B7 ligands reasonably well.

By comparing the dose-response curves for the two antibodies as predicted from our simulations, it is apparent that, at any given dose concentration, tremelimumab (Fig. 4b) is able to inhibit higher percentage of B7–1 and B7–2, when compared to that of ipilimumab (Fig. 4a). For instance, 1 μM of tremelimumab was found to inhibit ~ 54% of B7–1 binding and ~ 76% of B7–2 binding, which are much higher % inhibition than those obtained from 1 μM of ipilimumab (~ 25% of B7–1 inhibition; ~ 47% inhibition of B7–2) in our simulations. Such trends are in line with the affinity of these antibodies against CTLA-4 as reported by He et al [1]. It has been reported that both the antibodies have comparable association rate constants, however, the dissociation rate constants of ipilimumab is much higher than that of tremelimumab [1]. Hence, tremelimumab is able to block much higher amounts of B7 ligands from binding to CTLA-4, when matched with ipilimumab. In addition, it can be noted that higher percent inhibition of CTLA-4/B7–2 interactions than CTLA-4/B71 interactions can be achieved with low concentrations (≤ 10 μM) of the antibodies. This again accords with previous observation that the affinity of CTLA-4 to B7–1 is higher than its affinity to B7–2 [51]. In order to test this statement, we performed a simulation with our model, where only B7–1 and B7–2 were allowed to competitively bind with CTLA-4. To model this scenario, we again left the total initial concentrations of the antibodies and the CD28 receptor to 0, such that they do not have any effects on CTLA-4 binding to the B7 ligands. The result from this simulation is shown in Fig. 4c. Except for the first few minutes of the simulations, when the synapse was dominated by B7–2, the predominant amount of CTLA-4 remained in complex with B7–1 and the proportion of CTLA-4/B7–2 complex was meager.

### Effects of antibody-mediation on the overall complex formation

**Fig. 5:**
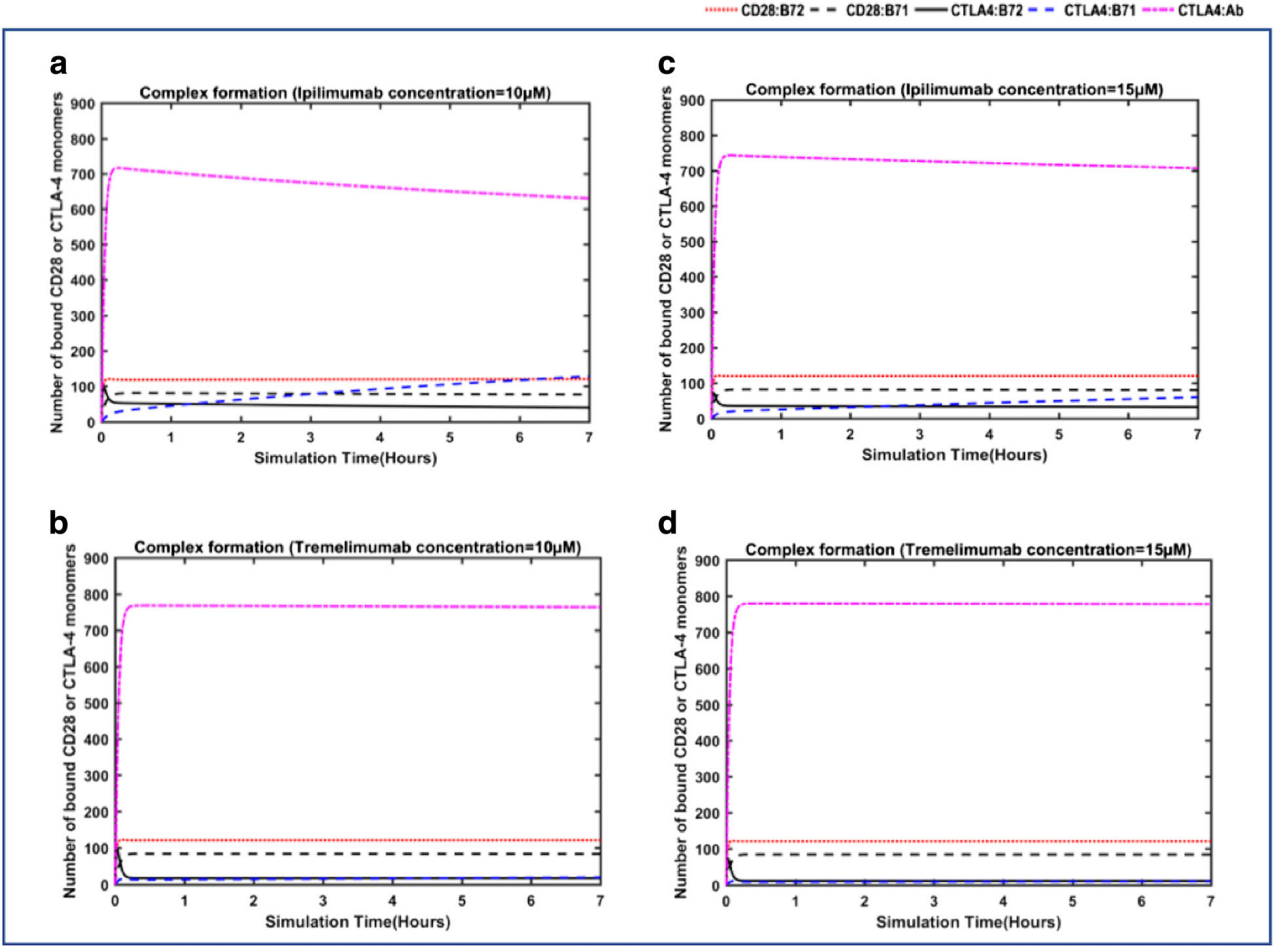
Antibody-mediated changes in the complex formation at the synapse in the free-diffusion model: The dynamic changes in the different complexes, such as CTLA-4/antibody, CTLA-4/B7 ligands and CD28/B7 ligands, at the synapse are simulated at 10 μM and 15 μM of antibodies, ipilimumab (**a**, **c**) and tremelimumab (**b**, **d**)

Subsequently, we tested the effects of antibody-mediation on the co-stimulatory (CD28/B7 ligands) and the co-inhibitory (CTLA-4/B7 ligands) complex formation at the synapse. Simulations were performed with a restraint-free competitive environment facilitated by the model, where all the species (such as CTLA-4, B7–1, B7–2 and CD28) were present and a specific concentration of either of the antibodies was added. Initially, 10 μM concentration of ipilimumab (Fig. 5a) and tremelimumab (Fig. 5b) were added in the simulations to study their effects. The results are comparable to Fig. 2a, where the simulation performed without the antibodies (concentration = 0 μM) is provided. As expected, upon addition of 10 μM concentration of antibodies, the antibody-bound CTLA-4 complex out-numbered the other complex formations. Particularly, in the antibody-free simulations, the B7–2-bound CTLA-4 complex (~ 600) was dominant during the initial hours; however, the presence of the antibody reduced the initial dominance of this complex (CTLA-4/B7–2) by at least 75%.

In the case of simulation with ipilimumab (concentration = 10 μM), it can be seen that both the B7 ligands and ipilimumab contested for CTLA-4 binding during the early hours. However, the antibody outcompeted the B7 ligands to form complexes with CTLA-4. It can be seen in Fig. 5a that, at the end of the simulations, there were ~ 630 CTLA-4/ipilimumab complexes against ~ 129 CTLA-4/B7–1 complexes. This is, particularly, a significant reduction in the sheer dominance of CTLA-4/B7–1 complexes (790 numbers) seen in the antibody-free simulations. On the other hand, 10 μM concentration of tremelimumab was able to bind more effectively with CTLA-4, thus remaining the most populated complex (~ 760 CTLA-4/tremelimumab complexes) at the steady state of the simulations, as shown in Fig. 5b. As a result, there were only 20 complexes of CTLA-4 and B7–1 at the end of simulations with tremelimumab (10 μM). In summary, the antibody-mediation has significantly impacted the CTLA-4/B7 ligand complex formation, which reduced from ~ 800 numbers (in antibody-free simulations) to ~ 168 and ~ 42 in the simulations with 10 μM of ipilimumab and tremelimumab, respectively. Later, we increased the dose of the antibodies to 15 μM and performed simulations (Fig. 5c-d). The higher dose of antibodies naturally increased the effects on CTLA-4 binding and B7-ligand blockade. In fact, predominant CTLA-4 monomers were engaged in complexes with the antibodies, ipilimumab (~ 707) and tremelimumab (~ 780). And as a result, the inhibitory complex formation between CTLA-4 and the B7-ligands had significantly diminished (~ 23 with 15 μM of tremelimumab; ~ 92 with 15 μM of ipilimumab). However, the addition of antibody did not significantly impact the interactions of CD28 with the B7 ligands. While the amount of CD28/B7–2 complexes remained almost the same in antibody-free and antibody-included simulations, the number of CD28/B71 complexes increased only slightly. The total numbers of different complexes (such as CTLA-4/mAb, CTLA-4/B7 and CD28/B7) at the end of simulations performed with different concentrations of ipilimumab and tremelimumab are summarized in Table 1.

**Table 1:** Comparison of the total numbers of CTLA-4/mAb, CTLA-4/B7 and CD28/B7 complexes formed at the end of simulations performed with the different concentrations (= 0 μM, 10 μM and 15 μM) of ipilimumab and tremelimumab

Interactions	Number of interacting CTLA-4/CD28 monomers					
	Initial	Antibody =0 μM	Tremelimumab		Ipilimumab	
			(10 μM)	(15 μM)	(10 μM)	(15 μM)
CD28/B7–2	0	127	121	121	121	120
CD28/B7–1	0	54	83	84	77	81
CTLA-4/B7–2	0	8	17	12	40	32
CTLA-4/B7–1	0	790	20	11	129	60
CTLA-4/mAb	0	0	763	778	631	707

**Fig. 6:**
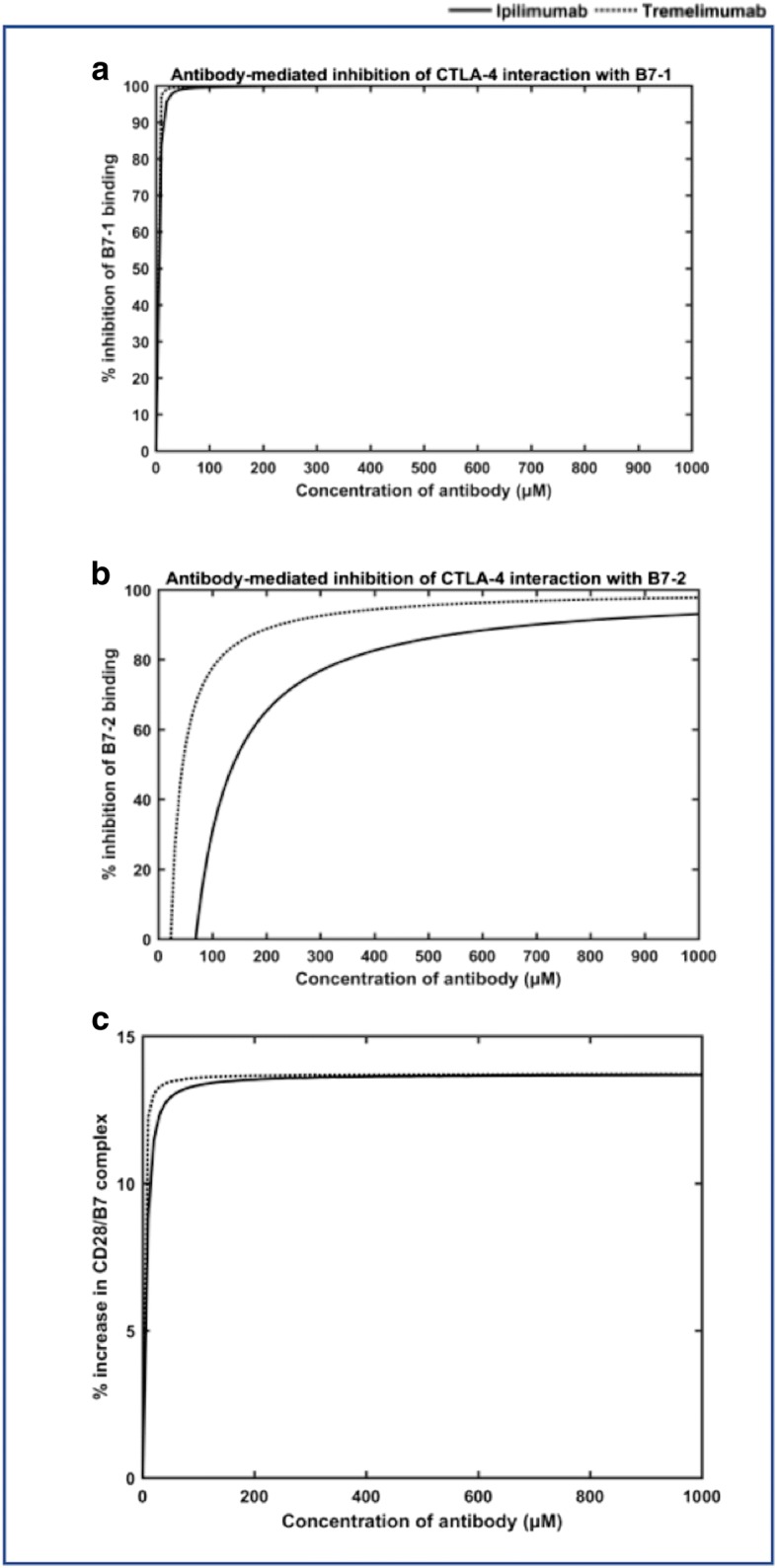
The simulation of full dose-response curves for the two antibodies, ipilimumab (**a**) and tremelimumab (**b**) in the free-diffusion model. The % increase in the CD28/B7 complex during the full-dose response simulations with ipilimumab and tremelimumab are also plotted (**c**)

Next, we simulated full dose-response curves for the two antibodies (Fig. 6), ipilimumab and tremelimumab. The inhibition percentage of the B7 ligands (i.e., B7–1 and B7–2) at each concentration of the antibodies in this figure was calculated as follows,

7 $$ \%B7\  inibition@x\ \mu M\  of\  mAb\kern0.5em =\left(1-\frac{\left( No. of\ CTLA-4/B7\  complex\right)@x\ \mu M\  of\kern0.5em mAb}{\left( No. of\ CTLA-4/B7\  complex\right)@0\ \mu M\  of\  mAb}\right)\times 100 $$

From the dose curves, it is clear that both the antibodies have effectively inhibited the bivalent interactions of CTLA-4 and B7–1 even at very small concentrations. In fact, 50% of the B7–1 interactions with CTLA-4 were inhibited at concentrations < 5 μM in the case of both the antibodies. As much as 10 μM of either of the antibodies was sufficient to achieve ~ 90% (or more) inhibition of CTLA-4/B7–1 interactions (Fig. 6a). These percentage inhibition values for CTLA-4/B7–1 interactions shown by the antibodies in the fully competitive simulations are almost similar to those observed for B7–2 knockout-simulations (where B7–2 and CD28 were absent, in Fig. 4). Nevertheless, the inhibition of B7–2 interactions (with CTLA-4), in the full model simulations, required really very high dose of the antibodies. In fact, it can be seen that the CTLA-4/B7–2 inhibition was seen only at concentrations ≥20 μM for Tremelimumab and > 60 μM for ipilimumab (Fig. 6b). This indicates that at lower concentrations of the antibodies, there were actually some increase in the CTLA-4/B7–2 complexes, when compared to those seen in the untreated (or antibody-free) simulations. It should be noted that, in the antibody-free simulations, the CTLA-4/B7–2 complex dominated during the initial stages until the bivalent CTLA-4/B7–1 complex suppressed the monovalent CTLA-4/B7–2 interactions to become the dominant complex (see in Fig. 2). Whereas, in the antibody-mediated simulations, at low dose concentrations, the antibodies are more proactive in blocking the multivalent CTLA-4/B7–1 interactions, which relieves the suppression on the monovalent CTLA-4/B7–2 interactions. In addition, the actual numbers of CTLA-4/B7–2 complexes are in general much less than that of CTLA-4/B7–1 complex. For example, at the end of antibody-free simulations, there were only 8 CTLA-4/B7–2 complexes, when compared to 790 CTLA-4/B7–1 complexes. Nevertheless, higher antibody concentrations effectively blocked the CTLA-4/B7–2 interactions as well. This contradicts with the B7–1 knockout simulations (Fig. 4), where even the lower amounts of the antibodies inhibited predominant CTLA-4/B7–2 interactions. This suggests that the competitive effects could implicate the dose response predictions significantly. Finally, as indicated earlier, inhibition of CTLA-4/B7 interactions did not essentially translate to the more proportional increase in the CD28/B7 complex formation. Figure 6c compares the percentage of increase in the CD28/B7 complex seen at each dose concentration (from 0 μM to 1000 μM) of ipilimumab and tremelimumab. It can be seen that there were only a maximum of ~ 14% gain in the CD28/B7 complexes following the inhibition of CTLA-4/B7 interactions by the mAbs in this study.

### Effects of antibody-mediation on the overall complex formation

**Fig. 7:**
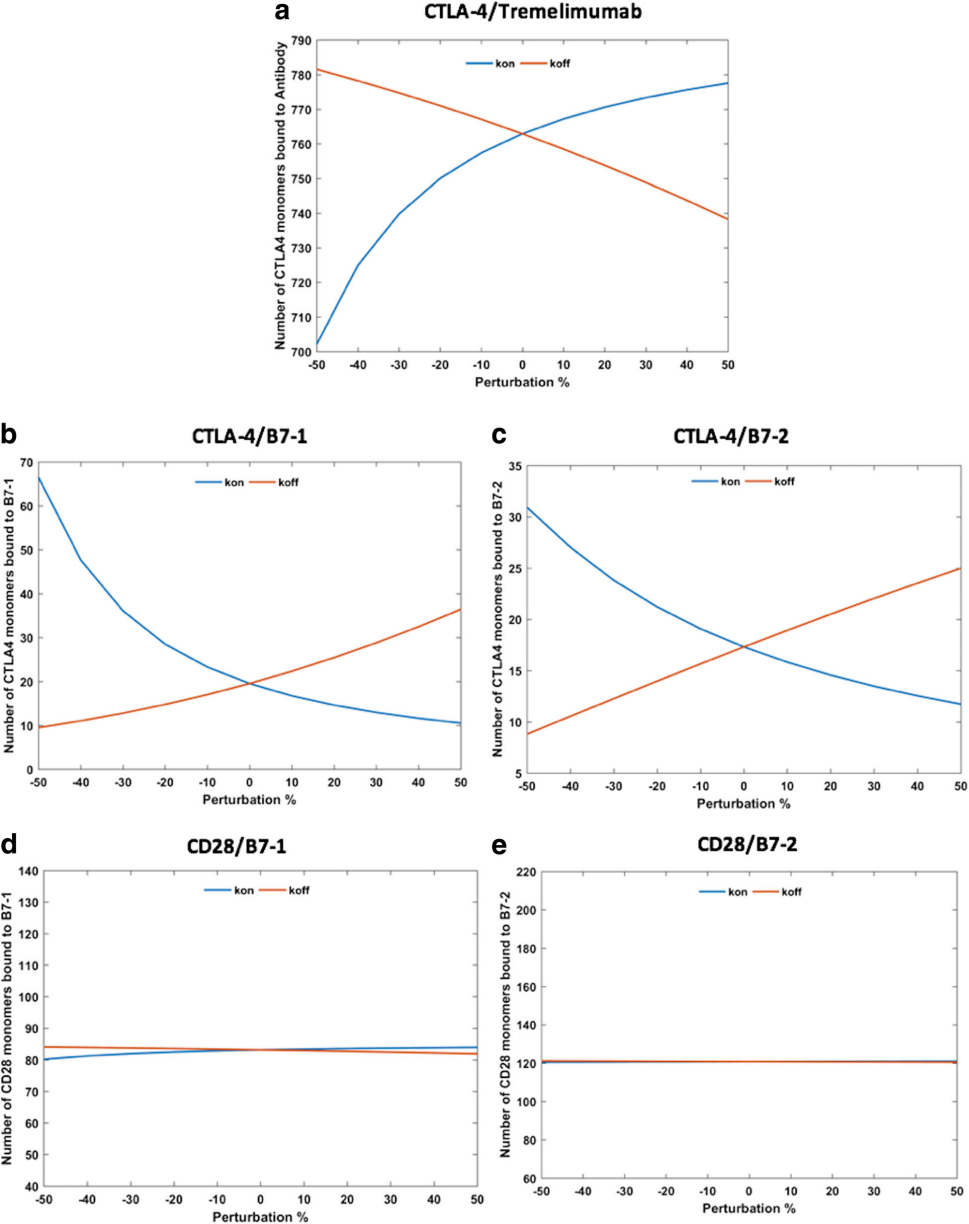
Sensitivity of the interactions towards the perturbations in the association (k_on_) and dissociation (k_off_) rate constants for CTLA-4/Tremelimumab complex. The changes in the numbers of CTLA-4/Tremelimumab complex (**a**), CTLA-4/B7–1 complex (**b**), CTLA-4/B7–2 complex (**c**), CD28/B7–1 complex (**d**), and CD28/B7–2 complex (**e**) in response to the perturbations are shown

It is important to acknowledge that any mathematical model is mainly dependent on the parameters employed to build it. Particularly, the sensitivity is much higher in the models that rely on biological parameters. For example, Jansson et al. [33] tested the dependence of their model on the affinity, mobility and expression levels of various species, by reducing each of these parameters by 10-fold, and found that some of the simulated interactions are sensitive to these changes [33]. However, it is true even in the case of measured data, as the experimental conditions, such as stoichiometry and temperature, have been shown to affect the results. For instance, an earlier experimental study reported that the affinity between CTLA-4 and B7–1 was 12 nM [30]; while another experimental study reported the affinity for the same complex to be 0.4 μM [31]. In the current study, we tested the sensitivity of our model towards the changes in the rate constants for association and dissociation of different species in the model (the parameters, P2-P20 listed in Additional file 1: Table S1). To achieve this, we perturbed the values for each of these parameters in the range of − 50 to + 50% from their respective original values used in the model. Figure 7 compares the effects of changing the association (k_on_) and dissociation (k_off_) rate constants for tremelimumab on different interactions, such as CTLA-4/antibody complex (Fig. 7a), CTLA-4/B7–1 (Fig. 7b), CTLA-4/B7–2 (Fig. 7c), CD28/B7–1 (Fig. 7d) and CD28/B7–2 (Fig. 7e). As expected, the CTLA-4/antibody and the CTLA-4/B7 interactions were the most affected by these perturbations; indeed, the effects seen for CTLA-4/antibody complex were inverse to those of CTLA-4/B7 interactions. For example, the 50-fold reduction in the k_on_ value led to the drop of ~ 60 numbers of monomer CTLA-4/antibody complexes, which was compensated by the increase in the total numbers of CTLA-4/B7 complexes (approximately + 48 for CTLA-4/B7–1 complex; + 12 for CTLA-4/B7–2 complex). On the other hand, 50-fold increase in the k_on_ value resulted in a small gain of CTLA-4/antibody complexes, which again led to the drop in the total numbers of CTLA-4/B7 complexes (refer to Fig. 7a-c). Similar inverse effects in the CTLA-4/antibody and CTLA-4/B7 complexes were also seen for the changes in the k_off_ values. Nevertheless, the CD28/B7 interactions were mostly insensitive to these perturbations. This highlights the fact that CD28 is not able to compete with the high-affinity and high-avidity interactions of CTLA-4/B7 and CTLA-4/antibody interactions.

The effects of varying the rate constants for association and dissociations for monovalent CD28/B7 complexes, monovalent CTLA-4/B7 complexes, bivalent CD28/B7–1 complex, and multivalent CTLA-4/B7 complexes were also tested (results provided in supplementary information, Additional file 4: Figure S1 and Additional file 5: Figure S2). These manipulated simulations described that the CTLA-4/antibody and CTLA-4/B7–1 interactions are mostly sensitive to the rate constant values for multivalent association and dissociation of CTLA-4/B7–1 complex; whereas, the CTLA-4/B7–2 interactions are predominantly affected by the rate constants for association and dissociation of bivalent CTLA-4/B7–2 interactions. The perturbations in the latter parameters also exhibited small effects on CTLA-4/antibody interactions. Nevertheless, none of the changes corresponding to CTLA-4/B7 interactions made any significant impacts on the CD28/B7 complexes, which were only sensitive to the rate constant values corresponding to their own association/dissociation. Although the model is sensitive to upto 50% variations in the selected kinetics parameters, the qualitative inference based on the original values remain the same.

## Discussions

Mathematical modeling and simulation remains a valuable tool to develop quantitative insights about the dynamic changes taking place within complex systems. It has particularly been employed in the field of cancer immunotherapy. Jansson et al. [33] developed a model for quantitative analysis of costimulatory complex formation, between CTLA4, CD28 and the B7- ligands, at the immunological synapse. However, there have been no study that modelled the effects of antibody-mediation on the complex formation at the synapse. In this study, we have taken one baby-step forward towards analyzing the effects of adding anti-CTLA-4 antibodies on the immunological balance between the co-stimulatory interactions (formed by CD28 and B7 ligands) and the co-inhibitory interactions (formed by CTLA-4 and B7 ligands) at the synapse, using a free-diffusion model. The study mainly focused on two promising CTLA-4 blocking antibodies, ipilimumab and tremelimumab, which are either in the market or in clinical trials, respectively. As acknowledged throughout the paper, this study is an extension of the Jansson’s model [33], where we included several new equations and parameters to account for the effects of antibody-mediation at the synapse. The model is able to reproduce the K_D_ values for the inhibition of CTLA-4 by the two antibodies. We also validated our model by showing a reasonable agreement between the dose-curves, for blocking the binding of B7–1 and B7–2 to CTLA-4 by ipilimumab, from our simulations against a previous experimental data from competitive binding assays by Keler et al. [50]. The study also helped to understand the relative efficacy of the two antibodies in CTLA-4 blockade. Although both tremelimumab and ipilimumab have similar K_D_ values, the former tends to show more effective inhibition of CTLA-4/B7 interactions, due to its much lower dissociation rate that that of ipilimumab. The modelling and simulations in this work have shown that different factors, such as multivalent interactions, mobility of molecules and competition effects, could impact the effects of antibody-mediation. The results, in particular, highlighted that the competitive effects played an important role in the dose-dependent inhibition of the B7 ligand interactions with CTLA-4 receptor by the antibodies. However, it is important to concede that, as in any case of mathematical modelling, the model in this work is also mainly dependent on the parameters employed to build it. However, it is known that the K_D_ values measured for the same systems under different experimental conditions could vary significantly. For example, different k_on_ and k_off_ rates for ipilimumab/CTLA-4 complex have been reported in the literature. Hence, in order to minimize the impacts from such variabilities, we have used the k_on_ and k_off_ rates for both ipilimumab/CTLA-4 and tremelimumab/CTLA-4 complexes from the same work of He et al. [1], which was very recently published.

Another important limitation of this model is that it is constructed based on the normal T-cell conditions, where both the co-stimulatory and co-inhibitory interactions at the synapse play important role in maintaining the much-needed immunological balance. However, a CTLA-4 blocking antibody (or any immune-checkpoint drug for that matter) is only administered in an abnormal micro-tumor environment, where the expression of the receptors and ligands will be different than those seen in the normal T-cells, thus shifting the balance more towards inhibitory interactions. But, unfortunately, comprehensive parameter data for simulating cancerous cells in the context of immunological synapse is not available in the literature. Hence, we made an informed choice of simulating the effects of antibody mediation in a normal T-cell environment, for which parameters are available and a preliminary model [33] (without antibody) was also published. Precisely, for this reason, we did not perform simulations by introducing the antibodies at various timescale (after reaching steady-state for instance). Instead, we only focused on simulating the competitive binding aspects of the antibodies to CTLA-4 and how it changes the co-stimulatory (CD28/B7) and the co-inhibitory (CTLA-4/B7) complex formations at the synapse, when compared to untreated (or antibody-free) simulation.

Despite the stated limitations, the numerical simulations performed with the current model are in agreement with different experiments, such as the dose curve for ipilimumab-mediated inhibition of B7 ligands. The model is able to predict the dose-dependent inhibition of CTLA-4/B7 interactions in an immunologically-relevant competitive environment, where both the B7-ligands and antibodies compete to bind with CTLA-4. In general, it is difficult (and not always practical) to measure the specific inhibition percentage of either B7–1 or B7–2 by the antibodies under such fully immunologically-relevant competitive binding environment. Most experiments measure the competitive binding of the anti-CTLA-4 antibodies only in the presence of either of the ligands and CTLA-4. Thus, this mathematical model could be a useful tool to gain some insights about the potencies of the antibodies to compete with both B7–1 and B7–2 to bind with the CTLA-4 receptor, at the dynamic immunological synapse. Although the simulations in this work were performed for only the two known antibodies, the model itself could serve as an easily transferable tool to study the effects of any anti-CTLA-4 antibodies on the co-stimulation by the CD28 pathway, provided the binding kinetics data for the query antibodies and CTLA-4 are available. Therefore, the results presented and the mathematical model will be useful for the research activity in the field of immune-checkpoints-targeted cancer therapy.

## Conclusion

In this work, we have developed an expanded mathematical modeling framework to quantitatively analyze the effects of anti-CTLA-4 antibody-mediation on the co-stimulatory and co-inhibitory complex formation at the immunological synapse. The numerical simulations performed using this model have been validated by different experimental data. The model predicted the dose curve for the B7-ligand blockade by ipilimumab, which was in a reasonable agreement with the experimental data obtained from competitive binding assays. Further, the model was also able to reproduce the K_D_ values for the binding of the antibodies against the CTLA-4 receptor. Our findings show that a number of significant factors, such as multivalent interactions, mobility of moleculesand competition effects contribute to the antibody-mediated interactions at the synapse. In particular, the competitive effects play a more predominant role. The simulations from our model show that in a less-competitive setting, the CTLA-4/B7–2 interactions are inhibited with much lower concentrations of antibodies, while the inhibition of B7–1 interactions required comparatively higher dose of antibodies. This is in concurrent with the previous findings that B7–1 is a preferred ligand for CTLA-4 and also has a higher affinity to CTLA-4 compared with B7–2. Nevertheless, our simulations show that the trend is reversed within a fully competitive and dynamic immunological synapse. In fact, the antibodies are more proactive in inhibiting the divalent CTLA-4/B7–1 interactions, which in turn relieves the suppression of CTLA-4/B7–2 complexes. As a result, the inhibition of CTLA-4/B7–2 in the full model required much higher concentrations of antibodies. Further, the inhibition of the CTLA-4/B7 interactions does not essentially lead to significant increase in the costimulatory CD28/B7 complexes. It is important to acknowledge that the model suffers from some of the important limitations, which are mainly caused due to lack of several parameters required to model a tumor microenvironment. Nevertheless, the current work represents an important first step towards understanding the antibody-mediated effects on synaptic complex formation. The model could also serve as an easily transferable predictive tool to study the effects of any anti-CTLA-4 antibodies on the co-stimulation by the CD28 pathway, provided the binding kinetics data for the query antibodies and CTLA-4 are available. Our natural next step will be expanding the current model by integrating it with the simulation of the main first signal (from TCR-MHC interactions) and also connecting to some downstream signaling processes, such as interleukin-2 activation pathway. Such an integrated mathematical model will be an excellent tool to guide immune-checkpoints research towards complete elimination of cancers.

## Additional files

Additional file 1: **Table S1.** Parameters used in the model. The parameters related to the interactions of CTLA-4 and CD28 with the B7 ligands in the current model are provided in this file. (DOCX 26 kb)

Additional file 2: **Table S2.** Equations related to the rate of change of density for the antibody included in different complexes. The rate of change of density for the different antibody-mediated complexes are given in this file. Equations related to the rate of change of density for the antibody included in different complexes. The rate of change of density for the different antibody-mediated complexes are given in this file. (DOCX 144 kb)

Additional file 3: **Table S3.** Different components/species included in the model and their respective abbreviation used in the text. The different components included in our mathematical model, along with their corresponding abbreviations, are provided in this file. Different components/species included in the model and their respective abbreviation used in the text. The different components included in our mathematical model, along with their corresponding abbreviations, are provided in this file. (DOCX 13 kb)

Additional file 4: **Figure S1.** Sensitivity of the CTLA-4/antibody (a, d) and CTLA-4/B7 (b-c, e-f) interactions towards the perturbations in the association and dissociation for the CTLA-4/B7 and the CD28/B7 complexes. This file includes various figures corresponding to the sensitivity analyses performed to study the impacts of perturbations in the association and dissociation rates for the CTLA-4/B7 and the CD28/B7 complexes. (DOCX 5236 kb)

Additional file 5: **Figure S2.** Sensitivity of the CD28/B7–1 and CD28/B7–2 interactions towards the perturbations in the association and dissociation for the CTLA-4/B7 and the CD28/B7 complexes. This file includes various figures corresponding to the sensitivity analyses performed to study the impacts of perturbations in the association and dissociation rates for the CTLA-4/B7 and the CD28/B7 complexes. (DOCX 181 kb)

## Abbreviations

APCs: Antigen-presenting cells
CTLA-4: Cytotoxic T-lymphocyte antigen 4
FDA: Food and drug administration
mAbs: Monoclonal antibodies
MHC: Major histocompatibility complex
ODEs: Ordinary differential equations
TCRs: T-cell receptors
