# Supplementary material for: A mathematical modelling tool for unravelling the antibody-mediated effects on CTLA-4 interactions
Source: BMC Med Inform Decis Mak. 2018 Jun 11;18:37. doi: 10.1186/s12911-018-0606-x (PMC5996525; doi:10.1186/s12911-018-0606-x)
Supplement: Supplementary file 1 — Table S1. Parameters used in the model. The parameters related to the interactions of CTLA-4 and CD28 with the B7 ligands in the current model are provided in this file. (DOCX 26 kb) [file 12911_2018_606_MOESM1_ESM.docx]

**Table S1.** Parameters used in the model

|  | Abbreviation | Description | Value | Reference |
| --- | --- | --- | --- | --- |
| P1 | a_syn_ | Area of the synapse | 12.6$\mu m$^2^ | ^1, 2^ |
| P2 | $\kappa$_28_ | Rate constant for CD28 diffusing out of synapse | 3.6*10^-2^s^-1^ | ^2-4^ |
| P3 | $\gamma$_28_ | Rate constant for CD28 diffusing into synapse | 1.0*10^-3^s^-1^ | ^2-4^ |
| P4 | $\lambda$ | Injection rate of CTLA-4 | 7.7*10^-3^s^-1^ | ^2, 5^ |
| P5 | $\kappa$_DC_ | Rate constant for B7-1 and B7-2 diffusing out of synapse | 2.7*10^-2^s^-1^ | ^2-4^ |
| P6 | $\gamma$_DC_ | Rate constant for B7-1 and B7-2 diffusing into synapse | 2.7*10^-4^s^-1^ | ^2-4^ |
| P7 | $\alpha$_1_ | Rate constant for association of CD28 and B7-2 | 0.77$\mu m$^2^ s^-1^ | ^2, 6, 7^ |
| P8 | $\alpha$_2_ | Rate constant for association of CD28 and B7-1 | 0.22$\mu m$^2^ s^-1^ | ^2, 6, 7^ |
| P9 | $\alpha$_3_ | Rate constant for association of CTLA-4 and B7-2 | 1.09$\mu m$^2^ s^-1^ | ^2, 6, 7^ |
| P10 | $\alpha$_4_ | Rate constant for association of CTLA-4 and B7-1 | 1.19$\mu m$^2^ s^-1^ | ^2, 6, 7^ |
| P11 | $\alpha$_22_ | Rate constant for bivalent association of CD28  and B7-1 | 0.22$\mu m$^2^ s^-1^ | ^2, 6, 7^ |
| P12 | $\alpha$_33_ | Rate constant for bivalent association of CTLA-4 and B7-2 | 0.13$\mu m$^2^ s^-1^ | ^2, 6, 7^ |
| P13 | $\alpha$_44_ | Rate constant for multivalent association of CTLA-4 and  B7-1 | 0.17$\mu m$^2^ s^-1^ | ^2, 6, 7^ |
| P14 | $\delta$_1_ | Rate constant for dissociation of CD28/B7-2 | 28 s^-1^ | ^2, 6, 7^ |
| P15 | $\delta$_2_ | Rate constant for dissociation of CD28/B7-1 | 1.6 s^-1^ | ^2, 6, 7^ |
| P16 | $\delta$_3_ | Rate constant for dissociation of CTLA-4/B7-2 | 5.1 s^-1^ | ^2, 6, 7^ |
| P17 | $\delta$_4_ | Rate constant for dissociation of CTLA-4/B7-1 | 0.43 s^-1^ | ^2, 6, 7^ |
| P18 | $\delta$_22_ | Rate constant for dissociation of bivalent CD28/B7-1 | 1.6 s^-1^ | ^2, 6, 7^ |
| P19 | $\delta$_33_ | Rate constant for dissociation of bivalent CTLA-4/B7-2 | 0.052 s^-1^ | ^2, 6, 7^ |
| P20 | $\delta$_44_ | Rate constant for dissociation of multivalent  CTLA-4/B7-1 | 0.0044 s^-1^ | ^2, 6, 7^ |
| P21 | t_CD28,tot_ | Total expression of CD28 | 9.2*10^3^ | ^2^ |
| P22 | t_CTLA,tot_ | Total expression of CTLA-4 | 0.4*10^3^ | ^2^ |
| P23 | d_B71,tot_ | Total expression of B7-1 | 2*10^3^ | ^2^ |
| P24 | d_B72,tot_ | Total expression of B7-2 | 43*10^3^ | ^2^ |
| P25 | m_CD28_ | Fraction of mobile CD28 | 0.3 | ^2, 8^ |
| P26 | m_B71_ | Fraction of mobile B7-1 | 0.6 | ^2, 8^ |
| P27 | m_B72_ | Fraction of mobile B7-2 | 0.6 | ^2, 8^ |
| P28 | m_CTLA_ | Fraction of mobile CTLA-4 | 1 | ^2, 8^ |
| P29 | i_B71_ | Immobile B7-1 molecules inside synapse | 0.64 $\mu m$^-2^ | ^2^ |
| P30 | i_B72_ | Immobile B7-2 molecules inside synapse | 13.7 $\mu m$^-2^ | ^2^ |
| P31 | i_CD28_ | Immobile CD28 molecules inside synapse | 14 $\mu m$^-2^ | ^2^ |

**References**

1. Grakoui, A.; Bromley, S. K.; Sumen, C.; Davis, M. M.; Shaw, A. S.; Allen, P. M.; Dustin, M. L., The Immunological Synapse: A Molecular Machine Controlling T Cell Activation. *Science* **1999**, 285, 221.

2. Jansson, A.; Barnes, E.; Klenerman, P.; Harlén, M.; Sørensen, P.; Davis, S. J.; Nilsson, P., A Theoretical Framework for Quantitative Analysis of the Molecular Basis of Costimulation. *The Journal of Immunology* **2005**, 175, 1575.

3. Agrawal, N. G. B.; Linderman, J. J., Mathematical Modeling of Helper T Lymphocyte/Antigen-presenting Cell Interactions: Analysis of Methods for Modifying Antigen Processing and Presentation. *Journal of Theoretical Biology* **1996**, 182, 487-504.

4. Szabo, A.; Schulten, K.; Schulten, Z., First passage time approach to diffusion controlled reactions. *The Journal of Chemical Physics* **1980**, 72, 4350-4357.

5. Egen, J. G.; Allison, J. P., Cytotoxic T Lymphocyte Antigen-4 Accumulation in the Immunological Synapse Is Regulated by TCR Signal Strength. *Immunity* **2002**, 16, 23-35.

6. Collins, A. V.; Brodie, D. W.; Gilbert, R. J. C.; Iaboni, A.; Manso-Sancho, R.; Walse, B.; Stuart, D. I.; van der Merwe, P. A.; Davis, S. J., The Interaction Properties of Costimulatory Molecules Revisited. *Immunity* **2002**, 17, 201-210.

7. van der Merwe, P. A.; Bodian, D. L.; Daenke, S.; Linsley, P.; Davis, S. J., CD80 (B7-1) Binds Both CD28 and CTLA-4 with a Low Affinity and Very Fast Kinetics. *The Journal of Experimental Medicine* **1997**, 185, 393-404.

8. Bromley, S. K.; Iaboni, A.; Davis, S. J.; Whitty, A.; Green, J. M.; Shaw, A. S.; Weiss, A.; Dustin, M. L., The immunological synapse and CD28-CD80 interactions. *Nat Immunol* **2001**, 2, 1159-1166.
