# Supplementary material for: A mathematical modelling tool for unravelling the antibody-mediated effects on CTLA-4 interactions
Source: BMC Med Inform Decis Mak. 2018 Jun 11;18:37. doi: 10.1186/s12911-018-0606-x (PMC5996525; doi:10.1186/s12911-018-0606-x)
Supplement: Supplementary file 2 — Table S2. Equations related to the rate of change of density for the antibody included in different complexes. The rate of change of density for the different antibody-mediated complexes are given in this file. Equations related to the rate of change of density for the antibody included in different complexes. The rate of change of density for the different antibody-mediated complexes are given in this file. (DOCX 144 kb) [file 12911_2018_606_MOESM2_ESM.docx]

**Table S2.** Equations related to the rate of change of density for the antibody included in different complexes.

| **AcA complex (i.e., antibody/(CTLA-4)_2_/antibody):** $\frac{dAcA}{dt}=k_{on}*Ac*Ab-k_{off}*AcA$  |
| --- |
| **Ab/CTLA4/B72 complex (i.e., mAb/(CTLA-4)_2_/B7-2):** $\begin{matrix} \frac{dAb/CTLA4/B72}{dt}=k_{on}*CTLA4/B72*Ab-k_{off}*Ab/CTLA4/B72 \\ +\alpha_{33}*Ac*B72-\delta_{33}*Ab/CTLA4/B72 \end{matrix}$  |
| **EAb_1_ complex (i.e., mAb/(CTLA-4)_2_/B7-1):** $\begin{matrix} \frac{dEAb_{1}}{dt}=k_{on}*E_{1}*Ab-k_{off}*EAb_{1}+\alpha_{44}*Ac*B71-\delta_{44}*EAb_{1} \\ -\alpha_{44}*EAb_{1}*CTLA4+\delta_{44}*CAb_{1} \end{matrix}$ $\begin{matrix} \frac{dEAb_{k+1}}{dt}=+k_{on}*Ab*E_{k+1}-k_{off}*EAb_{k+1}+\alpha_{44}*CAb_{k}*B71-\delta_{44}*EAb_{k+1} \\ -\alpha_{44}*EAb_{k+1}*CTLA4+\delta_{44}*CAb_{k+1} \end{matrix}$    |
| **DAb_k_ complex (i.e., Ab/((CTLA-4)_2_/B71(CTLA-4)_2_)_k_/Ab):** $\frac{dDAb_{k}}{dt}=+k_{on}*CAb_{k}*Ab-k_{off}*DAb_{k}$  |
| **CAb_k_ complex (i.e., ((CTLA-4)_2_/B71/(CTLA-4)_2_)_k_/Ab):** $\begin{matrix} \frac{dCAb_{k}}{dt}=-\alpha_{44}*CAb_{k}*B71+\delta_{44}*EAb_{k+1}+k_{on}*C_{k}*Ab-k_{off}*CAb_{k}+\alpha_{44}*EAb_{k}*CTLA4-\delta_{44}*CAb_{k} \\ -k_{on}*CAb_{k}*Ab+k_{off}*DAb_{k} \end{matrix}$  |
