# Supplementary material for: A mathematical modelling tool for unravelling the antibody-mediated effects on CTLA-4 interactions
Source: BMC Med Inform Decis Mak. 2018 Jun 11;18:37. doi: 10.1186/s12911-018-0606-x (PMC5996525; doi:10.1186/s12911-018-0606-x)
Supplement: Supplementary file 3 — Table S3. Different components/species included in the model and their respective abbreviation used in the text. The different components included in our mathematical model, along with their corresponding abbreviations, are provided in this file. Different components/species included in the model and their respective abbreviation used in the text. The different components included in our mathematical model, along with their corresponding abbreviations, are provided in this file. (DOCX 13 kb) [file 12911_2018_606_MOESM3_ESM.docx]

**Table S3.** Different components/species included in the model and their respective abbreviation used in the text. Note: CTLA-4, CD28 and B7-1 represent the corresponding homo dimers formed.

|  | **Components of the model** | **Abbreviation used** |
| --- | --- | --- |
| 1 | Unbound CD28 at synapse | CD28 |
| 2 | Unbound B7-1 at synapse | B71 |
| 3 | Unbound B7-2 at synapse | B72 |
| 4 | Unbound CTLA-4 at synapse | CTLA4 |
| 5 | CD28 outside the synapse | CD28out |
| 6 | B7-1 outside the synapse | B71out |
| 7 | B7-2 outside the synapse | B72out |
| 8 | Intracellular CTLA-4 | CTLA4int |
| 9 | CD28/B7-2 | CD28/B72 |
| 10 | CD28/B7-1 | CD28/B71 |
| 11 | CD28/B7-1/CD28 | (CD28)_2_/B71 |
| 12 | CTLA-4/B7-2 | CTLA4/B72 |
| 13 | B7-2/CTLA-4/B7-2 | CTLA4/(B72)_2_ |
| 14 | Antibody | Ab |
| 15 | CTLA-4/Ab | Ac |
| 16 | Ab/CTLA-4/Ab | AcA |
| 17 | (CTLA-4/B7-1)_k_ | E_k_ |
| 18 | (B7-1/CTLA-4/B7-1)_k_ | B_k_ |
| 19 | (CTLA-4/B7-1/CTLA-4)_k_ | C_k_ |
| 20 | Ab/(CTLA-4/B7-1)_k_ | EAb_k_ |
| 21 | Ab/(CTLA-4/B7-1/CTLA-4)_k_/Ab | DAb_k_ |
| 22 | Ab/(CTLA-4/B7-1/CTLA-4)_k_ | CAb_k_ |
| 23 | Ab/CTLA-4/B7-2 | Ab/CTLA4/B72 |
