# Supplementary material for: A mathematical modelling tool for unravelling the antibody-mediated effects on CTLA-4 interactions
Source: BMC Med Inform Decis Mak. 2018 Jun 11;18:37. doi: 10.1186/s12911-018-0606-x (PMC5996525; doi:10.1186/s12911-018-0606-x)
Supplement: Supplementary file 4 — Figure S1. Sensitivity of the CTLA-4/antibody (a, d) and CTLA-4/B7 (b-c, e-f) interactions towards the perturbations in the association and dissociation for the CTLA-4/B7 and the CD28/B7 complexes. This file includes various figures corresponding to the sensitivity analyses performed to study the impacts of perturbations in the association and dissociation rates for the CTLA-4/B7 and the CD28/B7 complexes. (DOCX 5236 kb) [file 12911_2018_606_MOESM4_ESM.docx]

**Figure S1**. Sensitivity of the CTLA-4/antibody (a, d) and CTLA-4/B7 (b-c, e-f) interactions towards the perturbations in the association and dissociation for the CTLA-4/B7 and the CD28/B7 complexes. The parameters named P7 to P20 numbered in the legends of the following figure corresponds to those listed in supplementary table, Table S1.


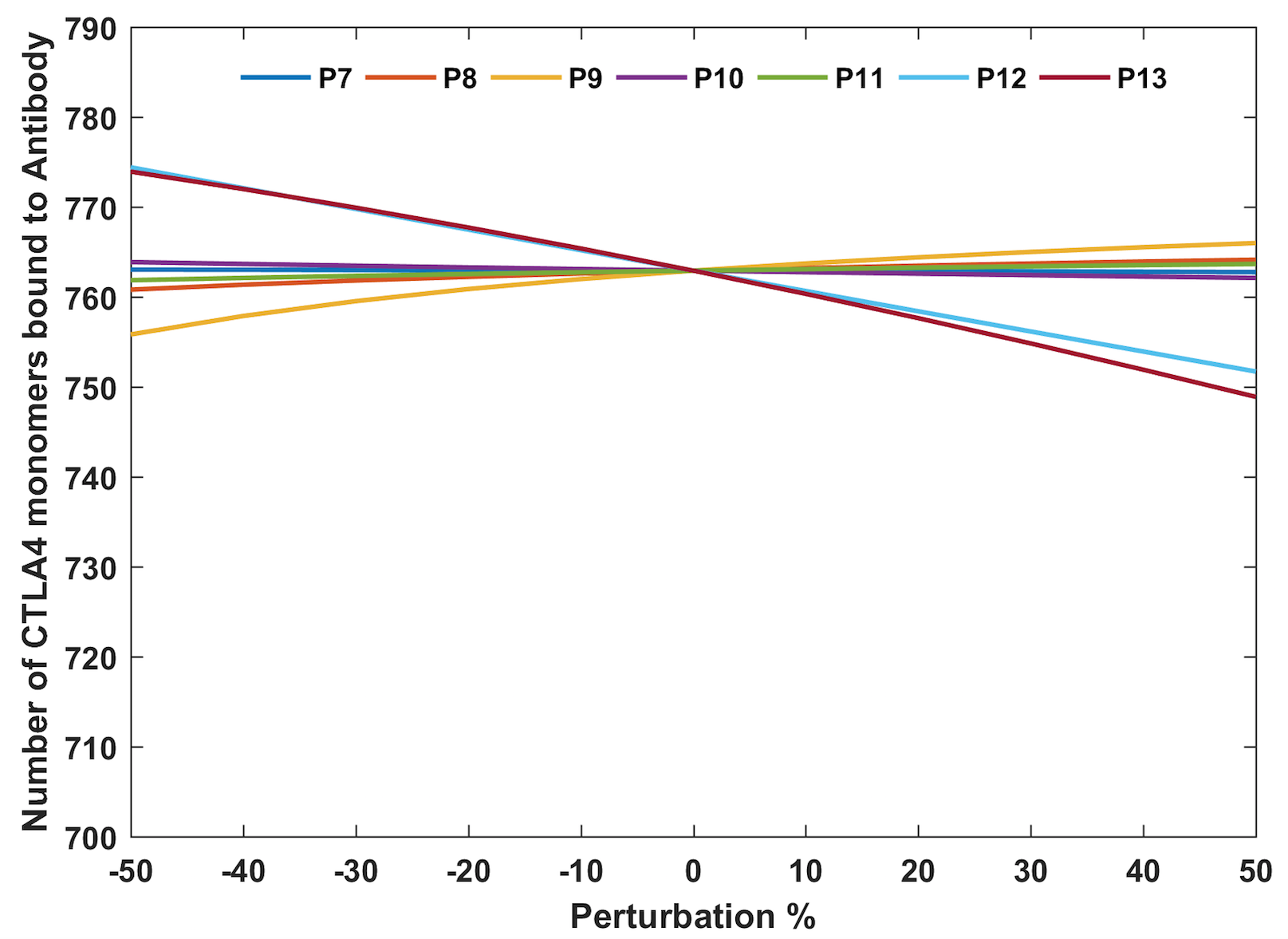

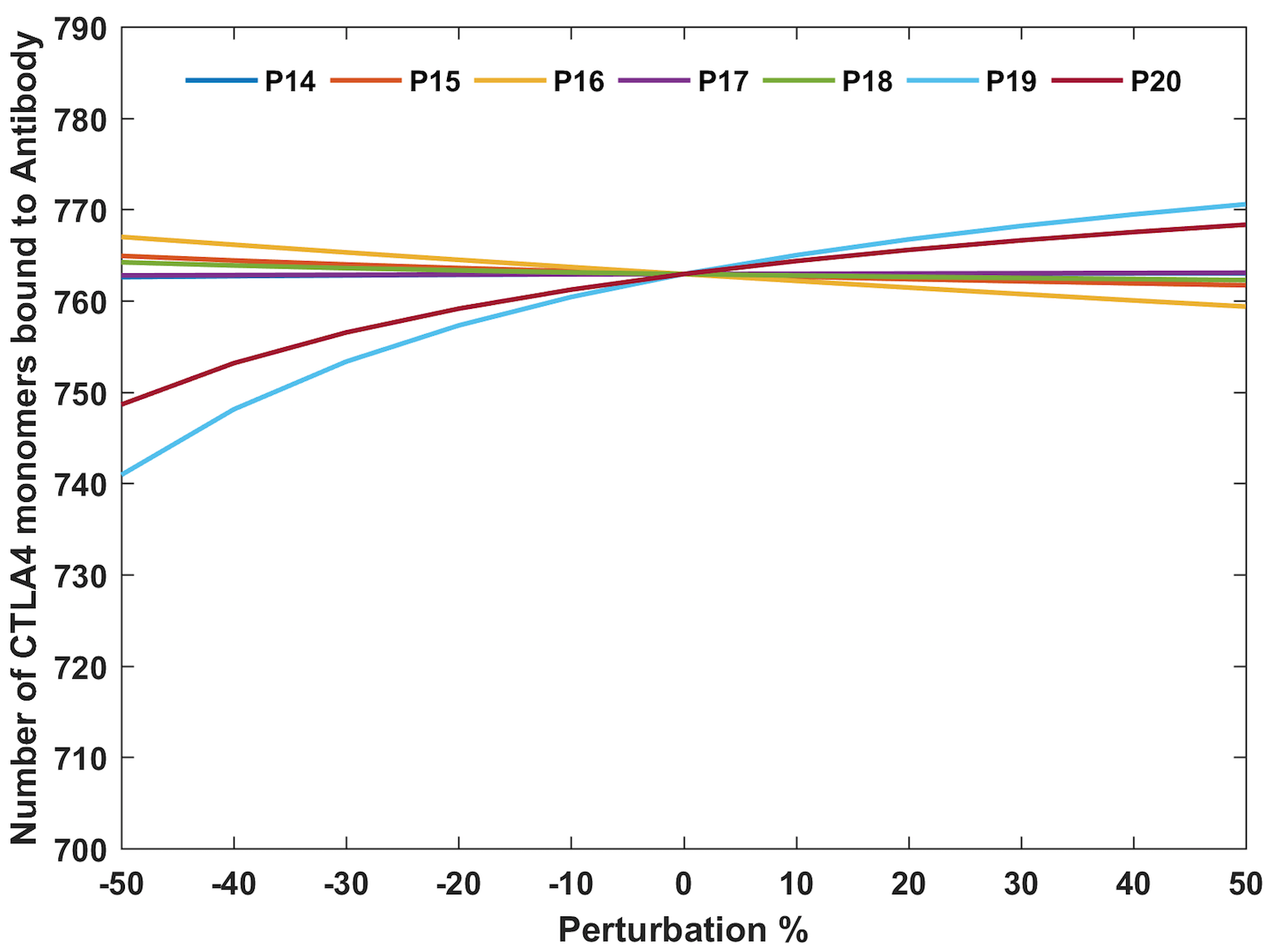


CTLA-4/Antibody


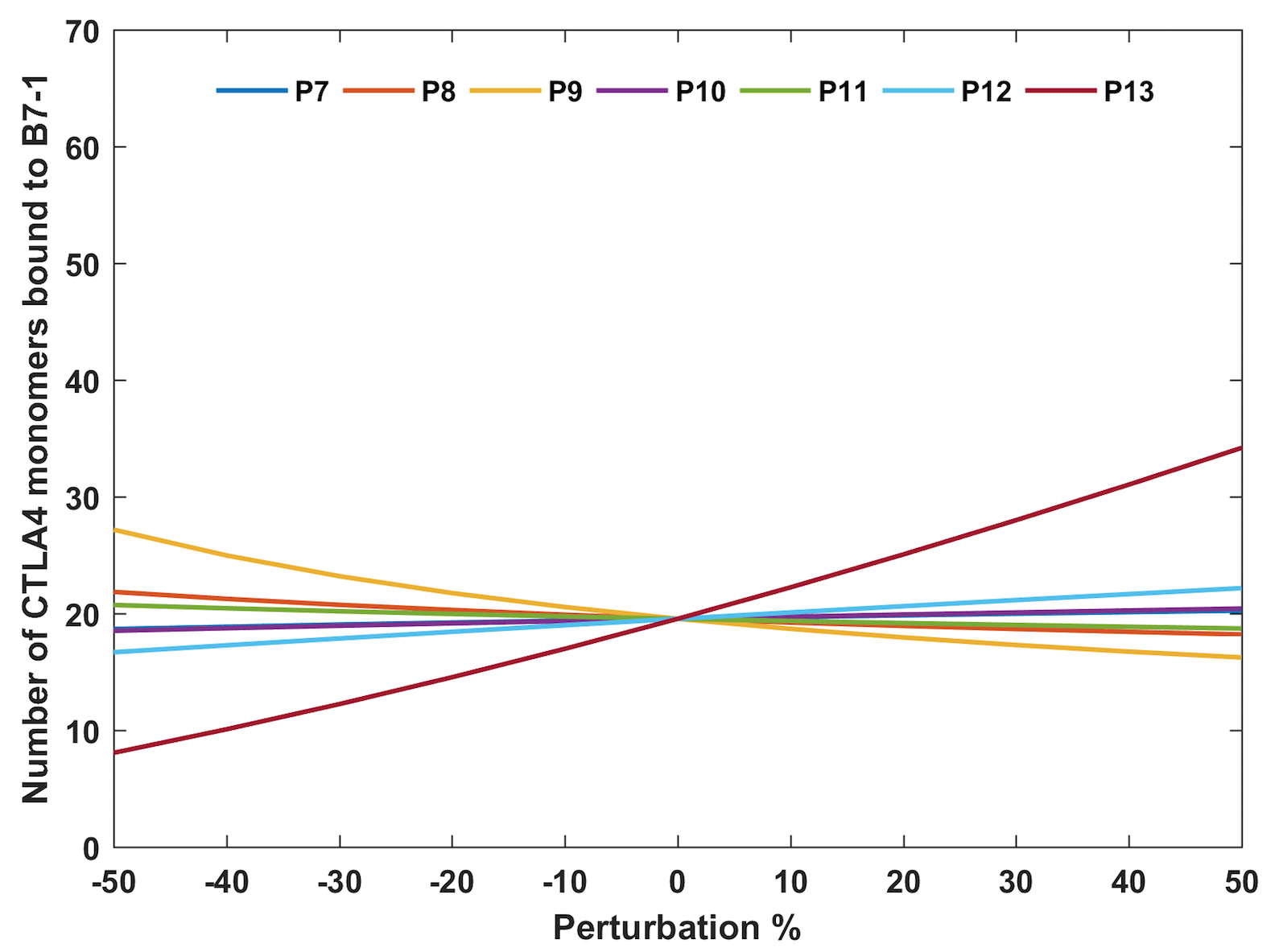

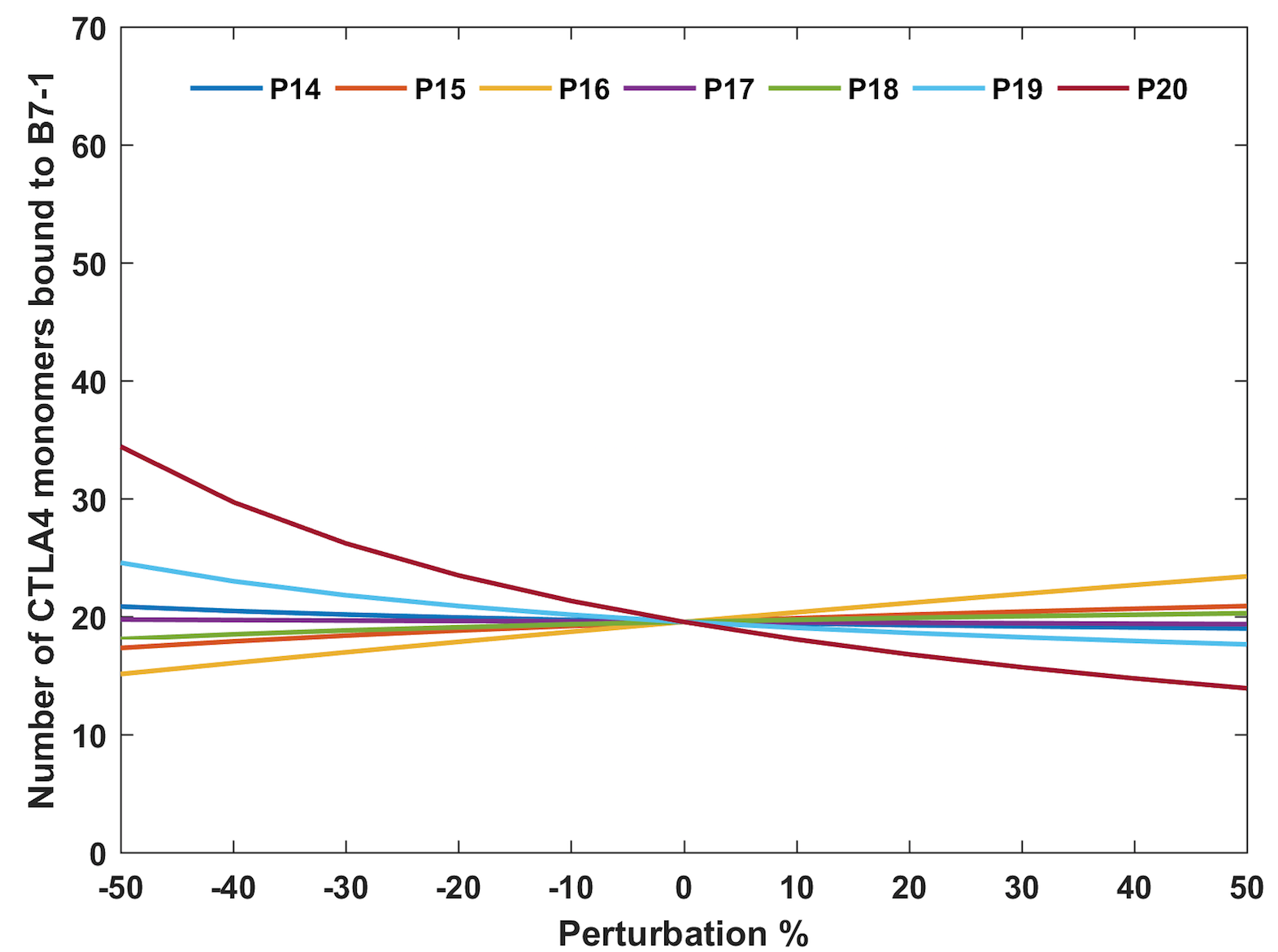

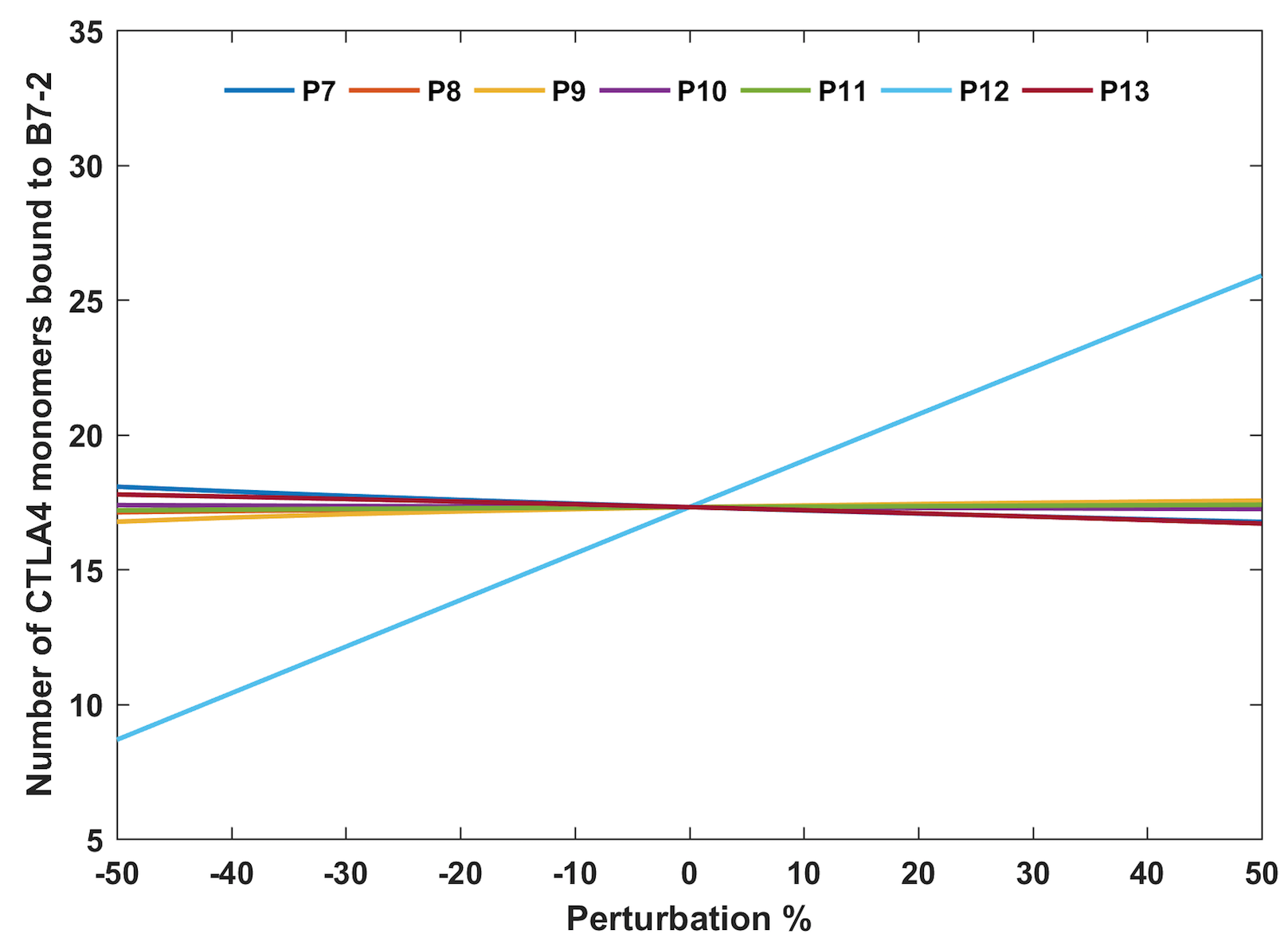

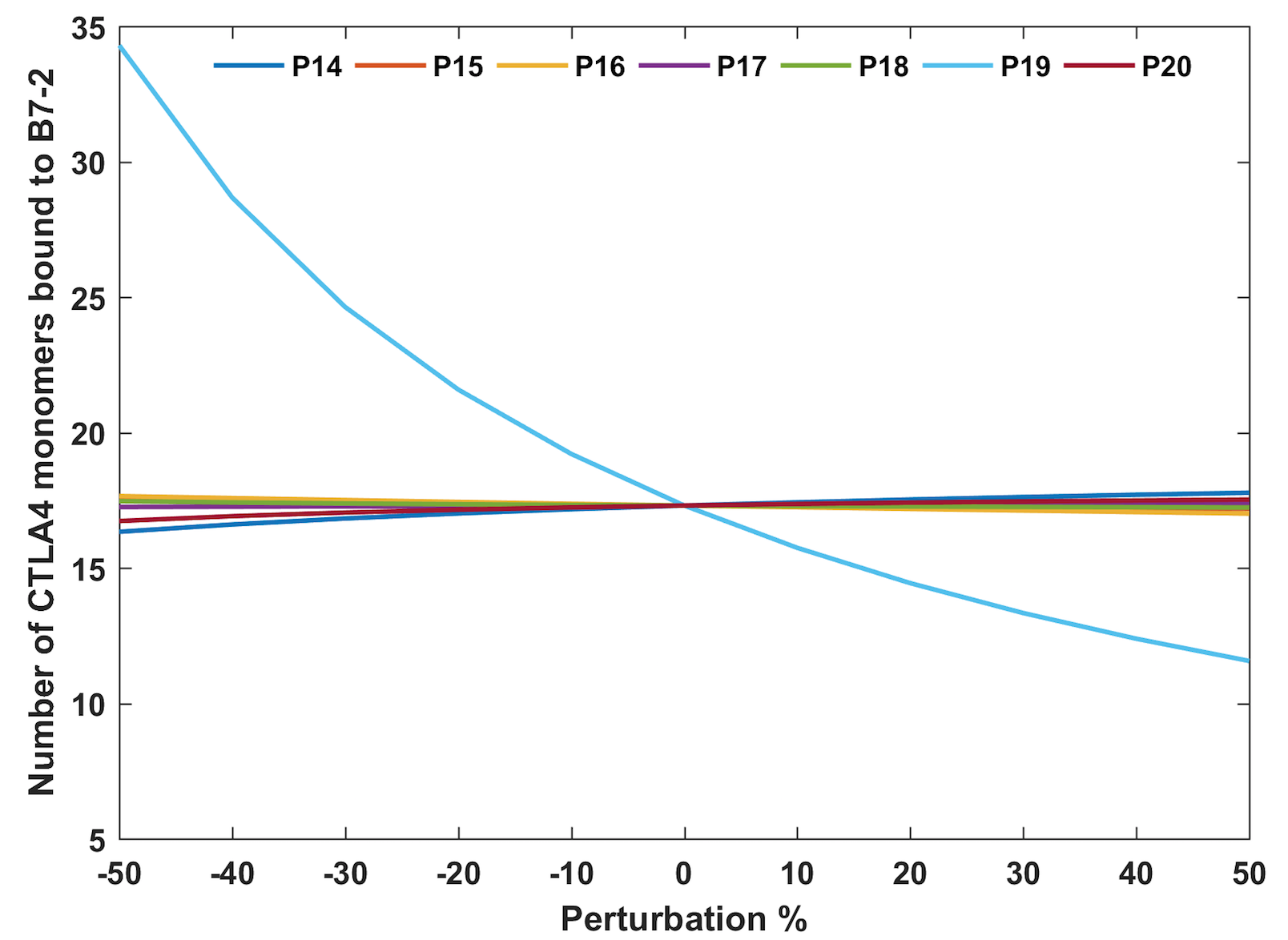


CTLA-4/Antibody

CTLA-4/B7-1

CTLA-4/B7-1

CTLA-4/B7-2

CTLA-4/B7-2

**(a)**

**(d)**

**(b)**

**(e)**

**(c)**

**(f)**
