# Supplementary material for: A mathematical modelling tool for unravelling the antibody-mediated effects on CTLA-4 interactions
Source: BMC Med Inform Decis Mak. 2018 Jun 11;18:37. doi: 10.1186/s12911-018-0606-x (PMC5996525; doi:10.1186/s12911-018-0606-x)

**Figure S2**. Sensitivity of the CD28/B7-1 (a-b) and CD28/B7-2 (c-d) interactions towards the perturbations in the association and dissociation for the CTLA-4/B7 and the CD28/B7 complexes. The parameters named P7 to P20 numbered in the legends of the following figure corresponds to those listed in supplementary table, Table S1.


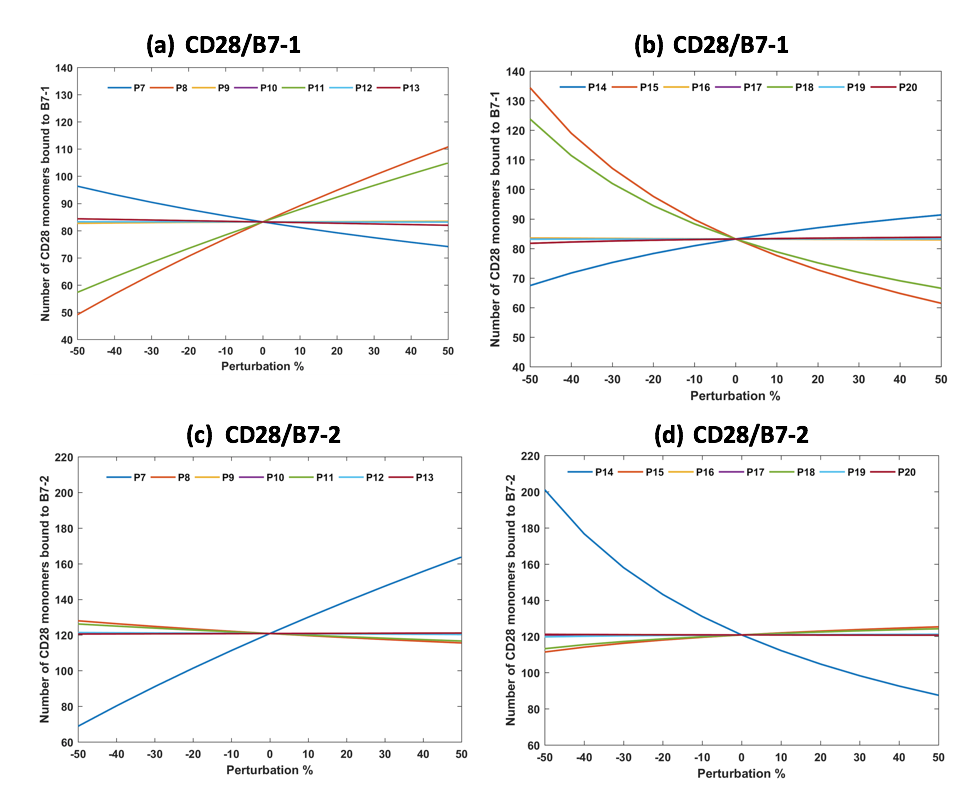

Supplement: Supplementary file 5 — Figure S2. Sensitivity of the CD28/B7–1 and CD28/B7–2 interactions towards the perturbations in the association and dissociation for the CTLA-4/B7 and the CD28/B7 complexes. This file includes various figures corresponding to the sensitivity analyses performed to study the impacts of perturbations in the association and dissociation rates for the CTLA-4/B7 and the CD28/B7 complexes. (DOCX 181 kb) [file 12911_2018_606_MOESM5_ESM.docx]
